# HNRNPK alleviates RNA toxicity by counteracting DNA damage in *C9orf72* ALS

**DOI:** 10.1007/s00401-022-02471-y

**Published:** 2022-07-27

**Authors:** Elke Braems, Valérie Bercier, Evelien Van Schoor, Kara Heeren, Jimmy Beckers, Laura Fumagalli, Lieselot Dedeene, Matthieu Moisse, Ilse Geudens, Nicole Hersmus, Arpan R. Mehta, Bhuvaneish T. Selvaraj, Siddharthan Chandran, Ritchie Ho, Dietmar R. Thal, Philip Van Damme, Bart Swinnen, Ludo Van Den Bosch

**Affiliations:** 1 Department of Neurosciences, Experimental Neurology and Leuven Brain Institute (LBI), KU Leuven-University of Leuven, Leuven, Belgium; 2 Center for Brain & Disease Research, Laboratory of Neurobiology, VIB, Campus Gasthuisberg, O&N5, Herestraat 49, PB 602, 3000 Leuven, Belgium; 3 Department of Imaging and Pathology, Laboratory of Neuropathology and Leuven Brain Institute (LBI), KU Leuven-University of Leuven, Leuven, Belgium; 4 Department of Neurosciences, Laboratory for Molecular Neurobiomarker Research and Leuven Brain Institute (LBI), KU Leuven-University of Leuven, Leuven, Belgium; 5 UK Dementia Research Institute, University of Edinburgh, Edinburgh, UK; 6 Centre for Clinical Brain Sciences, University of Edinburgh, Edinburgh, UK; 7 Cedars-Sinai Medical Center, Board of Governors Regenerative Medicine Institute, Los Angeles, CA USA; 8 Department of Pathology, University Hospitals Leuven, Leuven, Belgium; 9 Department of Neurology, University Hospitals Leuven, Leuven, Belgium

**Keywords:** *C9orf72*, ALS, RNA toxicity, HNRNPK, RRM2, DNA damage

## Abstract

A ‘GGGGCC’ repeat expansion in the first intron of the *C9orf72* gene is the most common genetic cause of amyotrophic lateral sclerosis (ALS) and frontotemporal dementia (FTD). The exact mechanism resulting in these neurodegenerative diseases remains elusive, but C9 repeat RNA toxicity has been implicated as a gain-of-function mechanism. Our aim was to use a zebrafish model for *C9orf72* RNA toxicity to identify modifiers of the ALS-linked phenotype. We discovered that the RNA-binding protein heterogeneous nuclear ribonucleoprotein K (HNRNPK) reverses the toxicity of both sense and antisense repeat RNA, which is dependent on its subcellular localization and RNA recognition, and not on *C9orf72* repeat RNA binding. We observed HNRNPK cytoplasmic mislocalization in *C9orf72* ALS patient fibroblasts, induced pluripotent stem cell (iPSC)-derived motor neurons and *post-mortem* motor cortex and spinal cord, in line with a disrupted HNRNPK function in *C9orf72* ALS. In *C9orf72* ALS/FTD patient tissue, we discovered an increased nuclear translocation, but reduced expression of ribonucleotide reductase regulatory subunit M2 (RRM2), a downstream target of HNRNPK involved in the DNA damage response. Last but not least, we showed that increasing the expression of HNRNPK or RRM2 was sufficient to mitigate DNA damage in our *C9orf72* RNA toxicity zebrafish model. Overall, our study strengthens the relevance of RNA toxicity as a pathogenic mechanism in *C9orf72* ALS and demonstrates its link with an aberrant DNA damage response, opening novel therapeutic avenues for *C9orf72* ALS/FTD.

**Supplementary Information:**

The online version contains supplementary material available at 10.1007/s00401-022-02471-y.

## Introduction

Amyotrophic lateral sclerosis (ALS) and frontotemporal dementia (FTD) are adult-onset neurodegenerative diseases belonging to a heterogeneous and complex disease spectrum [11, 71]. At one end of the spectrum, ALS is characterized by progressive decline of upper and lower motor neurons in the motor cortex, brainstem and spinal cord, leading to muscle weakness and wasting [10, 52]. Patients typically succumb to the disease within 2–5 years after diagnosis, mainly due to respiratory failure [10]. At the other end of the disease spectrum, changes in personality, social behavior and executive function are characteristic clinical features of FTD, which are associated with neurodegeneration in the frontal and temporal lobes of the brain [23, 52].

In ALS and FTD, impaired cognition often coincides with motor problems, and ubiquitinated inclusions containing transactive response DNA-binding protein 43 (TDP-43) represent a shared pathological hallmark [52, 69]. An explanation for this clinical and pathological overlap emerged with the discovery of a ‘GGGGCC’ hexanucleotide repeat expansion in the first intron of the *C9orf72* gene, which is the most common genetic cause of both disorders (C9 ALS/FTD) [16, 60]. It represents 40% and 25% of familial ALS and FTD cases respectively and accounts for a part of sporadic ALS/FTD patients as well [40, 59]. Patients have been reported to have from 38 to more than a thousand repeats, whereas unaffected individuals carry on average two to eight repeats [15, 17]. Remarkably, there is a large variability in clinical phenotypes, age at disease onset and disease progression of patients carrying a *C9orf72* (referred to as ‘C9’) repeat expansion [65], indicating the involvement of environmental and genetic modifying factors [1, 70].

The exact mechanism underlying C9 ALS/FTD remains elusive. However, three non-mutually exclusive mechanisms have been proposed and are currently being investigated using in vitro and in vivo ALS models in addition to patient *post-mortem* brain and spinal cord samples. A first mechanism attributes neurodegeneration to a *C9orf72* loss-of-function, due to impairment of normal *C9orf72* gene expression by the C9 repeat expansion [9]. In a second mechanism, called dipeptide repeat (DPR) toxicity, repeat-associated non-ATG (RAN) translation leads to the formation of five different DPR proteins (i.e., polyGA, polyGP, polyGR, polyPR, polyPA) [19]. These DPRs originate from C9 repeat sense and antisense transcripts, which are formed through bidirectional transcription of the hexanucleotide repeat sequence [19]. A third mechanism, coined RNA toxicity, is based on the sequestration of RNA-binding proteins (RBPs) by C9 repeat RNA into RNA foci, thereby interfering with normal RBP cellular functions such as splicing and transcriptional regulation [66]. Of all three mechanisms, RNA toxicity remains the least explored, which might be related to the difficulty in separating RNA from DPR toxicity, as DPRs are formed upon translation of C9 repeat RNA [66].

It is important to elucidate the contribution of RNA toxicity in C9 ALS/FTD since RBPs, shown to interact with sense and antisense *C9orf72* hexanucleotide repeat RNAs, are candidate modifying proteins of interest in the modulation of C9 ALS/FTD heterogeneity [12, 18, 25]. Indeed, recent studies confirmed the modifying potential of some of these proteins in *C9orf72* ALS [5, 47, 64]. For example, decreased expression of the nuclear export factor *ALYREF* in a C9 *Drosophila* model alleviates toxicity, whereas downregulation of *HNRNPA3* in primary rat hippocampal neurons and patient fibroblasts was found to aggravate the *C9orf72* ALS phenotype [5, 47]. As both RNA foci and DPRs are simultaneously present as pathological hallmarks in the majority of *C9orf72* models [47] and because RBPs have also been reported to interact with DPRs [32, 78], it is unclear whether RNA toxicity is at the root of the altered neurodegenerative phenotypes.

In this study, we aimed to assess the relevance of C9 RNA toxicity by identifying RBPs capable of modifying a repeat RNA-induced phenotype. Therefore, we used our previously characterized zebrafish model in which the *C9orf72* hexanucleotide repeat-associated ALS phenotype is only due to RNA toxicity, without any involvement of DPRs [64]. In addition, we already showed that overexpression of the transcriptional activator Purα prevented the C9 repeat RNA-induced motor axonopathy in these zebrafish [64]. By performing a targeted screen of candidate modifiers, we now discovered heterogenous nuclear ribonucleoprotein K (HNRNPK) as another potent suppressor of the repeat RNA-induced axonopathy. Using the *C9orf72* zebrafish model, as well as patient fibroblasts, iPSC-derived motor neurons and *post-mortem* material, we provide evidence that HNRNPK loss-of-function contributes to disease in *C9orf72* ALS. We identified the DNA damage and repair-related protein ribonucleotide reductase regulatory subunit M2 (RRM2) as a downstream target and mediator of HNRNPK dysregulation in ALS/FTD. In conclusion, we show that C9 repeat RNA toxicity is associated with an aberrant DNA damage response through disturbed RRM2 functionality. More specifically, we further emphasize the relevance of C9 repeat RNA toxicity as a disease mechanism by demonstrating the presence of DNA damage and diminished DNA repair in C9 ALS/FTD. Both hallmarks represent a novel avenue of research on C9 ALS/FTD and are exigent topics in the investigation of general ALS/FTD-related neurodegeneration.

## Materials and methods

### DNA constructs

Plasmids, comprising 91 (91S) or 70 (70S) ‘GGGGCC’ hexanucleotide repeats under control of a T7 promoter were obtained and adapted as described earlier [46, 64]. In the 70S plasmid, a reverse complementary sequence of the T3 promoter was inserted 30 bp after the repeats to generate antisense transcripts (70AS). GFP control plasmid construct (PS100010, Origene, Rockville, USA) was digested with *Age*I (ER1461, Thermo Scientific, Waltham, US) restriction enzyme. FLAG-tagged HNRNPK (RC201843, Origene) and RRM2 (RC228362, Origene) plasmid constructs were digested with *Age*I.

HNRNPK deletion constructs were synthesized by Genscript (Piscataway, USA) in a pUC57 vector and subcloned in a pCMV6-Entry vector (PS100001, Origene). After *Xho*I and *Eco*RI restriction digestion of donor constructs, desired inserts were harvested using the GenElute Gel Extraction kit (Sigma, Saint-Louis, US), according to the manufacturer’s instructions. Next, inserts were ligated (16 °C, overnight) with T4 ligase into the pCMV6 vector. These constructs were then transformed in *E. coli* TOP10 chemically competent cells (C404010, Invitrogen, Waltham, US). Consequently, plasmids were linearized with *Age*I restriction enzyme.

### In vitro RNA transcription and biotinylation

Following linearization of plasmids by restriction digestion, 91S repeat RNA, RBP RNA and HNRNPK deletion construct RNA was synthesized using mMESSAGE mMACHINE^®^T7 kit (Invitrogen). Antisense repeat (70AS) RNA was generated using mMESSAGE mMACHINE^®^T3 kit (Invitrogen). Resulting RNA was purified with MEGAclear Transcription Clean-Up Kit (Invitrogen) and quality was evaluated on a 1–2% RNA gel based on expected transcript lengths. RNA concentration was determined by spectrophotometry (Nanodrop, Thermo Scientific).

One-step RNA transcription and biotinylation of constructs containing 91 and 70 repeats was performed as previously described [42], using T7 RNA polymerase (P2075, Promega, Madison, US) for the production of biotinylated 91S repeat RNA and T3 RNA polymerase (P2083, Promega) for the production of biotinylated 70AS repeat RNA.

### Zebrafish maintenance and microinjections

Zebrafish work was performed as previously described [33]. All experiments were approved by the Ethics Committee of the KU Leuven (P112/2021). Overall, microinjection of the specified amounts of RNA or morpholino oligos was done in 1–2 cell stage zebrafish oocytes (as indicated in figure legends). Zebrafish embryos were kept in a 28.5 °C incubator. For Western blot, 6 h post fertilization (hpf) blastulae were collected after manual dechorionation and deyolking using forceps. For other purposes, zebrafish embryos were manually dechorionated using forceps at 24 hpf. Upon systematic visual inspection, only morphologically normal fish were retained for further analysis. Prior to fixation at 30 hpf, deyolking of embryos was accomplished using a glass Pasteur pipette. For RNA extraction, zebrafish embryos were collected on dry ice and stored at − 80 °C. For immunohistochemistry, zebrafish embryos were fixed overnight at 4 °C in 4% paraformaldehyde (PFA). Fixed embryos were stored in phosphate-buffered saline (PBS) at 4 °C.

Antisense morpholino oligonucleotides were designed and obtained from Gene Tools (Philomath, US): morpholino standard control oligo (sequence 5ʹ-CCTCTTACCTCAGTTACAATTTATA-3ʹ), *hnRNPK* splice-blocking oligo (5ʹ-AACTCAAAGACTTACTTTGCTCTGT-3ʹ), *hnRPKL* splice-blocking (5ʹ-ACATTTACATCACACTTACTTTGCT-3ʹ), *hnRNPK* translation-blocking oligo (5ʹ-GATCCATTATCTATCTACACCTAGT-3ʹ) and *hnRPKL* translation-blocking oligo (5ʹ-CTGCTCAATTTCTGTCTCCATTGTC-3ʹ).

### SV2 immunohistochemistry and analysis of axonal phenotype

Fixed zebrafish embryos were permeabilized with acetone for 1 h at − 20 °C, blocked with 1% bovine serum albumin (BSA)/1% dimethyl sulfoxide (DMSO)/PBS for 1 h at room temperature (RT) and immunostained with primary mouse anti-synaptic vesicle glycoprotein 2 (SV2, 1/200 in 1%DMSO/PBS, Developmental Studies Hybridoma Bank (Iowa City, US), Supplementary table 1, online resource) for 5 h at RT. Embryos were incubated overnight at 4 °C with secondary Alexa Fluor 555 anti-mouse antibody (1/500 in 1% DMSO/PBS, Invitrogen). In-between washing steps were performed with 1%BSA/1%DMSO/PBS and following SV2 staining, samples were washed 4 times in 0.2% PBS-Triton (PBST) for 1 h and stored in PBS at 4 °C.

Stained zebrafish embryos were analyzed with fluorescence microscopy (Leica DM 3000 LED microscope, 20x objective, DMK 33UX250 USB3.0 monochrome industrial camera, The Imaging Source, Wetzlar, Germany) using LUCIA software (version 4.60, Laboratory Imaging). A standardized method was used to score fish on axonal length and abnormal branching of ventral root projections. Consecutively, 15 fish were analyzed per condition. For axonal length scoring, five predefined, consecutive motor axons (i.e., the 8th up to the 12th axon on one side) were measured in all 15 embryos. Data were normalized to the control condition. Correspondingly, abnormal branching was analyzed from 20 predefined and consecutive motor axons (i.e., the 8th up to the 17th axon on both sides) of 15 embryos. Branching was considered to be abnormal when at least two out of 20 axons were branched at or above the ventral edge of the notochord. The number of biological replicates is indicated with “N” in the figure legends of the results section. For RNA injections, GFP mRNA-injected embryos were used as negative controls.

### Whole-mount zebrafish γH2AX immunohistochemistry and analysis

Fixed zebrafish embryos were permeabilized with acetone for 1 h at − 20 °C, blocked with 10% NDS in 0.4% PBST for 1 h at 37 °C and immunostained with primary rabbit anti-γH2AX (1/500 in 0.4% PBST, GeneTex (Irvine, US), Supplementary table 1, online resource) for 3 h at 37 °C. Embryos were incubated overnight at 4 °C with secondary Alexa Fluor 555 anti-rabbit antibody (1/1000 in 0.4% PBST, Invitrogen). In-between washing steps were performed with 0.4% PBST. Nuclei were stained using NucBlue counterstaining (Invitrogen) and embryos were mounted using ProLong Gold antifade reagent (Invitrogen). For image acquisition, a Nikon NiE microscope was used with a 40x objective, equipped with a Yokogawa CSU-X spinning-disk module and Teledyne Photometrics Prime 95B camera. The setup was controlled by NIS-Elements (software version 5.30.05, Nikon Instruments Europe B.V.). For image analysis, the number of neuronal nuclei positive for γH2AX and located in the spinal cord of zebrafish embryos was counted and normalized to the length of the spinal cord section using ImageJ.

### Cell culture

Human Embryonic Kidney 293 (HEK) cells (ATCC-CRL-1573, LGC Standards GmbH, Wesel, Germany), HeLa cells (ATCC-CRM-CCL-2, LGC Standards GmbH, Wesel, Germany) and human-derived fibroblasts (control lines 3/2 and 3/3, *C9orf72* ALS patient lines 24/4 and 24/5) were grown and maintained at 37 °C with 5% CO2 in Dulbecco’s Modified Eagle Medium/Nutrient Mixture F-12 (DMEM/F-12) with high glucose and L-Glutamine (Gibco, Waltham, US) supplemented with 10% fetal bovine serum (FBS, Gibco), 5% non-essential amino acids (NEAA, Gibco) and penicillin/streptomycin (100 µg/ml). For transfection purposes, antibiotics were not added to media. Primary human fibroblasts were obtained from skin biopsies of ALS patients and controls with the approval of the Ethical Committee of the University Hospitals Leuven (S50354). Clinical information is indicated in Supplementary table 2 (online resource). Human *C9orf72* ALS patient iPSC lines and their isogenic controls were obtained and were published before [61]. Maintenance of iPSCs and motor neuron differentiation were performed as previously described [24].

### Transfection and siRNA-mediated knockdown of HEK and HeLa cells

Transfection of HEK cells with HNRNPK constructs was performed using Opti-MEM™ (Gibco) and Lipofectamine™ 2000 Transfection Reagent (Invitrogen) according to the manufacturer’s instructions. Cells of 6-well plates were lysed using RIPA buffer (Sigma) supplemented with protease inhibitor cOmplete™ (Roche, Basel, Switzerland) and collected 48 h post-transfection.

In HeLa cells, siRNA was reverse transfected using Opti-MEM™ (Gibco) and Lipofectamine™ RNAiMAX Transfection Reagent (Invitrogen) following the standard protocol. SiRNAs were obtained from Dharmacon (Lafayette, US): ON-TARGETplus human non-targeting siRNA (D-001810-01-05, target sequence 5ʹ-UGGUUUACAUGUCGACUAA-3ʹ), HNRNPK SMARTpool L-011692-00-0005 targeting J-011692-05 (5ʹ-UAAACGCCCUGCAGAAGAU-3ʹ), J-011692-06 (5ʹ-GGUCGUGGCUCAUAUGGUG-3ʹ), J-011692-07 (5ʹ-UGACAGAGUUGUUCUUAUU-3ʹ) and J-011692-08 (5ʹ-GCAAGAAUAUUAAGGCUCU-3ʹ). Cells were treated, collected or fixed at 72 h post-transfection.

### Camptothecin treatment of HeLa cells

Cultured cells were treated with 10 µM camptothecin (CPT) topoisomerase I inhibitor for 30 min. Then, medium was replaced with fresh medium (without drugs) and cells were incubated for 4 h at 37 °C before collection or cells were immediately PFA-fixed or lysed after treatment.

### Human autopsy cases

Brain tissue was collected in accordance with the applicable laws in Belgium (UZ Leuven) upon written informed consent. The study was approved by the UZ Leuven Ethical Committee (S65097, S59292, S60803, Leuven, Belgium). After autopsy, the right hemisphere was dissected into coronal planes and frozen at − 80 °C. The left hemisphere was fixed in 4% PFA. The *post-mortem* diagnosis of ALS and FTLD-TDP was pathologically confirmed by assessment of the pTDP-43 pathology. Clinical and neuropathological information is indicated in Supplementary table 3 (online resource).

### Western blot

RIPA buffer supplemented with protease inhibitor was used to lyse 6 hpf zebrafish blastulae, HEK cells and iPSC-derived motor neurons. For *post-mortem* material, 50 mg of frozen brain tissue was weighed and mechanically homogenized in 0.5 ml 2% SDS in Tris-buffered saline (TBS) with Nuclease (Pierce™ Universal Nuclease, Thermo Scientific) and a cocktail of protease/phosphatase inhibitors (Halt, Thermo Scientific) using a micropestle. Samples were sonicated, followed by a centrifugation at 14 000*g* for 30 min. The resulting supernatant was used. Protein quantification was done using (Micro) BCA™ Protein Assay Kit (Thermo Scientific). Indicated amounts of lysates were supplemented with Pierce™ Non-Reducing Sample Buffer (Thermo Scientific), loaded on 4–20% Mini-PROTEAN^®^ TGX Stain-Free™ Precast Gels (Bio-Rad, Hercules, US) and transferred to Trans-Blot^®^ Turbo™ Mini 0.2 µm PVDF Transfer Packs (Bio-Rad) using the Trans-Blot^®^ Turbo™ Transfer System (Bio-Rad). After blocking with 5% non-fat dry milk in TBS supplemented with 0.1% TWEEN^®^20 (Sigma) (TBS-T) for 1 h at room temperature, membranes were incubated overnight at 4 °C with primary antibody diluted in TBS-T. Primary antibodies are listed in Supplementary table 1 (online resource). Then, three TBS-T washes and a 1 h incubation with secondary antibody (polyclonal goat anti-rabbit/mouse-immunoglobulins/HRP, DAKP, 1/5000 in T-BST) at room temperature were performed before washing two times with TBS-T and once with TBS. Blots were visualized by chemiluminescence using SuperSignal™ West ECL/Pico/Femto Chemiluminescent Substrate (Thermo Scientific) on the ImageQuant™ LAS 4000 (GE Healthcare Life Sciences, Piscataway, US).

### RNA pulldown

HEK cells were transfected with HNRNPK deletion constructs using Lipofectamine 3000 (Invitrogen), according to the manufacturer’s instructions. WT and 48 h post-transfected HEK cells were lysed in RIPA buffer supplemented with protease inhibitor and RNasin^®^ Ribonuclease Inhibitor (Promega) by physical disruption using mortar and pestle. Magnetic streptavidin-coated beads (Life Technologies, Gent, Belgium) were prepared for RNA application, according to the manufacturer’s instructions. Next, HEK lysates were precleared for 1 h at 4 °C by incubation with magnetic beads on rotor. Non-specific binding was reduced by pre-incubating magnetic beads with precleared WT HEK lysate. Biotinylated 91S or 70AS repeat RNA was denatured at 95 °C for 10 min and incubated with 0.5 mg of precleared lysate overnight at 4 °C on rotor. The following day, 20 µl of prepared magnetic beads was added and incubated for 1 h at 4 °C on rotor. Samples were washed five times in ice-cold PBS, then denatured in Pierce™ Non-Reducing Sample Buffer (Thermo Scientific) and separated by SDS-PAGE followed by Western blotting.

### RT-PCR

Total RNA of 30 hpf zebrafish embryos was isolated using the RNeasy kit (Qiagen, Hilden, Germany). cDNA was generated using reverse transcription with SuperScript™ III First-Strand Synthesis SuperMix kit (Invitrogen). RT-PCR was performed using DreamTaq Green PCR Master Mix (Thermo Scientific) in a 96-well thermal cycler (Thermo Scientific) with optimized program, according to the manufacturer’s instructions. RT-PCR products were analyzed on a 2% agarose gel and visualized using MIDORI Green staining (Nippon Genetics, Düren, Germany). Primers were obtained from Integrated DNA Technologies (IDT, Leuven, Belgium): *hnRNPK* (forward 5ʹ-AAACGACCCACAGGAAGAGG-3ʹ, reverse 5ʹ-GCACTCCTGAAAGAGCTTGATG-3ʹ), *hnRPKL* (forward 5ʹ-GAACAGGCATTCAAACGCTC-3ʹ, reverse 5ʹ-CTCTGCCTCTCATTGGGAAC-3ʹ), *GAPDH* (forward 5ʹ-CCCATGTTTGTCATGGGTGT-3ʹ, reverse 5ʹ-GGTTGCTGTAACCGAACTCA-3ʹ).

### qPCR

Total RNA extraction was performed with the RNeasy Mini kit (Qiagen). cDNA was synthesized from 1 μg of total RNA using SuperScript III™ First-Strand Synthesis SuperMix (Invitrogen), according to the manufacturer’s instructions. Quantitative PCR was performed using SYBR Green PCR Master Mix (Applied Biosystems, Waltham, US) on the 7500 Step OnePlus Real-Time PCR system (Applied Biosystems). Relative gene expression was determined by the 2^−ΔΔct^ method and normalized to *ZNF48* transcript levels. The following primers (IDT) were used: *HNRNPK* (forward 5ʹ-GGTGGCTCCGGATATGATTATT-3ʹ, reverse 5ʹ-TGGTCCTGTGTTCCTGTAATG-3ʹ), *ZNF48* (forward 5ʹ-CAAACACCAGCGGACTCATA-3ʹ, reverse 5ʹ-TGCCACATTCACCACAGATAG-3ʹ).

### Immunocytochemistry

After culturing cells (HEK, HeLa, fibroblasts and iPSC-derived motor neurons) on a coverslip, cells were washed with PBS and PFA-fixed for 20 min. Three PBS wash steps were followed by 1 h blocking at 37 °C with 10% normal donkey serum (NDS) in 0.4% PBS-Triton (PBS-T). Then, cells were incubated with primary antibody diluted in 0.4% PBS-T overnight at 4 °C. Primary antibodies are listed in Supplementary table 1 (online resource). The next day, three 0.4% PBS-T wash steps were followed by 1 h incubation with secondary antibody conjugated with Alexa Fluor 555 or Alexa Fluor 488 (Invitrogen, 1/2000 in 0.4% PBS-T) at RT. Upon three 0.4% PBS-T washes, NucBlue™ Live ReadyProbes™ Reagent (Invitrogen) was added as a nuclear marker and after 20 min, cells were washed three times with PBS. Finally, coverslips were mounted on microscope slides using fluorescence mounting medium (Dako, Agilent, Santa Clara, US). Confocal images were obtained using the Leica SP8 DMI8 confocal microscope using a 40x objective. Images were processed using ImageJ.

### *Post-mortem* immunohistochemistry

To assess localization of HNRNPK in *post-mortem* brains, immunohistochemical analysis was performed on 7–8 µm thick sections cut from frozen tissue of motor cortex, as previously described [20]. Frozen tissue was used to circumvent antigen retrieval issues. Slides were washed with BondTM Wash Solution (Leica Biosystems, Nußloch, Germany) for 15 min at room temperature and stained overnight with HNRNPK primary antibody (Supplementary table 1, online resource) at 4 °C. The next day, slides were washed three times for 5 min with BondTM Wash Solution before incubation with Rabbit Alexa555 secondary antibody for 90 min at room temperature. Following three 5-min washing steps with BondTM Wash Solution (Leica), slides were washed once with PBS. To quench autofluorescence of lipofuscin, slides were incubated with TrueBlack^®^ (1/20 in 70% EtOH, Biotium, Fremont, US) for 30 s at room temperature. After rinsing three times with PBS, slides were mounted using ProLong™ Gold Antifade reagent with DAPI (Invitrogen). Confocal images were obtained using a Leica SP8 DMI8 confocal microscope. Images were processed using ImageJ.

To assess localization of RRM2 in *post-mortem* brain and spinal cord, histological examination was performed on 5 µm-thick sections cut from formalin-fixed, paraffin-embedded tissue. Stainings were performed with the BOND-MAX automated IHC/ISH Stainer (Leica Biosystems). Slides were deparaffinized and epitopes were retrieved with low pH buffer. After incubation with Envision Flex Peroxidase-Blocking Reagent, slides were incubated with a primary anti-RRM2 antibody (Supplementary table 1, online resource) for 30 min, followed by secondary antibody incubation. 3,3’-Diaminobenzidine (DAB) was used for visualization. Counterstaining with hematoxylin and dehydration was carried out in an autostainer, followed by mounting with an automated cover-slipper (Leica Biosystems). Images were acquired using the Leica DM2000 LED microscope using a 40x objective coupled to a Leica DFC 7000 T camera. Images were processed using ImageJ. Clinical and neuropathological information is indicated in Supplementary table 3 (online resource).

### Quantification of HNRNPK mislocalization and RRM2 translocation

On ImageJ, nuclei of immunofluorescent-stained HeLa cells, human-derived fibroblasts, iPSC-derived motor neurons and *post-mortem* tissue were selected in the DAPI channel and corresponding regions of interest (ROIs) were placed in an unbiased manner. Cytoplasmic ROIs were captured for the selected nuclei in the HNRNPK or RRM2 channel, respectively. HNRNPK/RRM2 nuclear and cytoplasmic ROI intensity was measured as the raw integrated density and was presented as the nuclear cytoplasmic (N/C) ratio. Each data point represents the average N/C ratio per replicate.

### RRM2 scoring in *post-mortem* brain and spinal cord

The presence of RRM2 in the DAB-stained nuclei of neurons in motor cortex, occipital cortex and cervicothoracic spinal cord was assessed in non-neurodegenerative controls and *C9orf72* ALS cases. A qualitative grading system was used in which cells were counted as positive when nuclei were stained completely and negative when nuclei were not stained for RRM2.

### Quantification of γH2AX foci

γH2AX foci were automatically quantified using a computational algorithm based on uniform threshold per fluorescence channel in ImageJ as described by the light microscope core facility at Duke university (https://microscopy.duke.edu/guides/count-nuclear-foci-ImageJ). The prominence in the ImageJ function “Find Maxima” was set to at least 40.

### RNA-sequencing

RNA-sequencing was performed by the Nucleomics Core Facility (VIB, Leuven, Belgium). RNA was isolated using the RNeasy kit (Qiagen). From extracted RNA, libraries were made using the Illumina TruSeq Stranded mRNA Library protocol. These libraries were sequenced on an Illumina NextSeq 500 paired-end 75 bp and yield an average of 37.5 million reads per sample (range 35.1–40.5). To estimate the expression of the transcript of every sample, reads were counted using Salmon (v0.14.0) [55] against ensemble transcript for the zebrafish reference genome GRCz11. Transcript level expression from the protein coding genes was summarized to the gene level expression using the R-package tximport (v1.12.0). Differential expression was performed with edgeR (v3.24.3) [43]. Genes with a FDR-adjusted *P* value smaller than 0.05 and with an altered expression of 30% were deemed significantly differentially expressed. Datasets are deposited and available in ENA (project accession PRJEB50765).

### Statistical analysis

Statistical analysis was performed using GraphPad Prism software (GraphPad Software v8.4, San Diego, US) or RStudio (v1.1.463, RStudio Team (2018). RStudio: Integrated Development for R. RStudio, Inc., Boston, US (http://www.rstudio.com)). Shapiro–Wilk normality test (GraphPad Prism) was used to assess normal distribution of data. For zebrafish experiments, axonal length and branching was analyzed with one-way ANOVA (GraphPad Prism) and Tukey’s post hoc correction was applied for multiple comparisons. Data are represented as mean ± SEM. Data from experiments with *post-mortem* material, iPSC-derived motor neurons, fibroblasts, HEK and HeLa cells are represented as mean ± SEM, unless indicated otherwise. Normally distributed data were analyzed using one-way ANOVA with Tukey’s post hoc correction for multiple comparisons or unpaired t tests for comparisons of two groups, unless indicated otherwise. Data that were not normally distributed were analyzed using Kruskal–Wallis non-parametric test with Dunn’s correction for multiple comparisons or Mann–Whitney *U* test for comparisons of two groups. Significance level was defined at 0.05. Significance levels are indicated in the figures as follows: *P < 0.05, **P < 0.01, ***P < 0.001, ****P < 0.0001.

## Results

### HNRNPK prevents toxicity in the *C9orf72* RNA zebrafish model

**Fig. 1:**
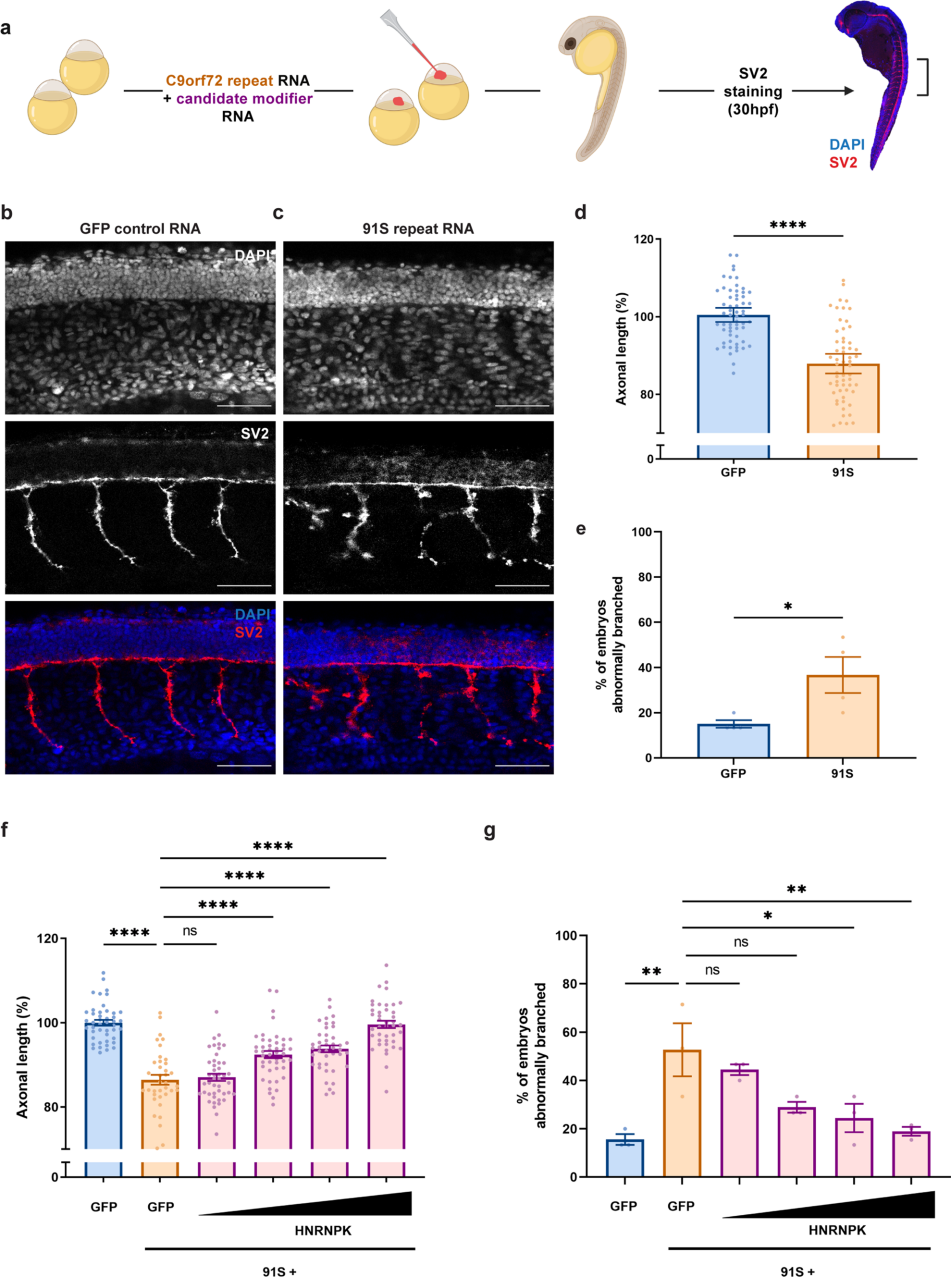
HNRNPK prevents the repeat RNA-induced axonopathy in a transient zebrafish model specific for *C9orf72* RNA toxicity. **a** Illustration of the *C9orf72* RNA toxicity zebrafish model. **b**, **c** SV2 immunostaining of motor axons in 30 hpf zebrafish embryos injected with GFP control mRNA (**b**) and 91 *C9orf72* hexanucleotide repeats in sense direction (91S) (**c**). Scale bar = 50 µm. **d**, **e** Percentage of axonal length compared to control (**d**) and percentage of embryos with abnormal branching (**e**) in 30 hpf zebrafish embryos injected with GFP control and 91S repeat RNA at equimolar dose (0.844 µM) (*N* = 4 experiments). Data represent mean ± SEM. Statistical significance was evaluated with unpaired *t* test; *P < 0.05, ****P < 0.0001. **f**, **g** Effect of HNRNPK mRNA injection (0.071-0.142-0.284-0.568 µM) on the 91S repeat RNA-induced axonopathy showing a dose-dependent rescue of axonal length (**f**) and abnormal branching (**g**) (*N* = 3 experiments). Data represent mean ± SEM. Statistical significance was evaluated with one-way ANOVA and Tukey’s multiple comparison test; *P < 0.05, **P < 0.01, ****P < 0.0001

To identify novel modifying factors of C9 ALS/FTD, we performed a targeted screen starting from RBPs previously shown to bind sense and/or antisense C9 repeat RNA [12, 25]. We used a zebrafish model specifically showing C9 RNA toxicity without the production of DPRs, as we demonstrated before [64]. Therefore, we micro-injected RNA containing 91 copies of human *C9orf72* hexanucleotide repeat in the sense direction (91S) in 1–2 cell stage zebrafish oocytes and observed motor axon abnormalities in zebrafish embryos (30 hpf) after whole-mount staining of synaptic vesicle glycoprotein 2a (SV2) (Fig. 1a–e), similar to the axonopathy induced in mutant SOD1 and TDP-43 zebrafish models [31, 33, 73]. In particular, reduced motor axonal length and increased aberrant branching were detected in *C9orf72* zebrafish compared to embryos injected with a *GFP* control mRNA encoding the GFP fluorescent protein. Next, we co-injected 91S repeat RNA with RNA encoding candidate modifying RBPs and analyzed the effect on axonal length and branching.

Following a dose–response experiment (Supplementary Fig. 1b, c, online resource), we injected the highest non-toxic dose of candidate RNA in zebrafish oocytes and found that, from all tested candidates, heterogeneous nuclear ribonucleoprotein K (HNRNPK) is the most potent modifier of *C9orf72* RNA toxicity in this model. More specifically, overexpression of HNRNPK, as confirmed by Western blot (Supplementary Fig. 1a, online resource), rescued the axonal phenotype in a dose-dependent manner (Fig. 1f, g). As HNRNPK is known to bind to cytidine-rich regions [8], we also assessed its potential modifying effect on C9 antisense (‘GGCCCC’) RNA toxicity [64]. Analogous to what we saw for sense repeat RNA toxicity, HNRNPK alleviated the motor axonopathy induced by antisense repeat RNA, consisting of 70 copies of the *C9orf72* hexanucleotide repeat in the antisense direction (70AS) (Supplementary Fig. 1d, e, online resource). These results show that HNRNPK is a strong modifier of C9 RNA toxicity in our zebrafish model.

### hnRNPK is essential for neuronal development in zebrafish

**Fig. 2:**
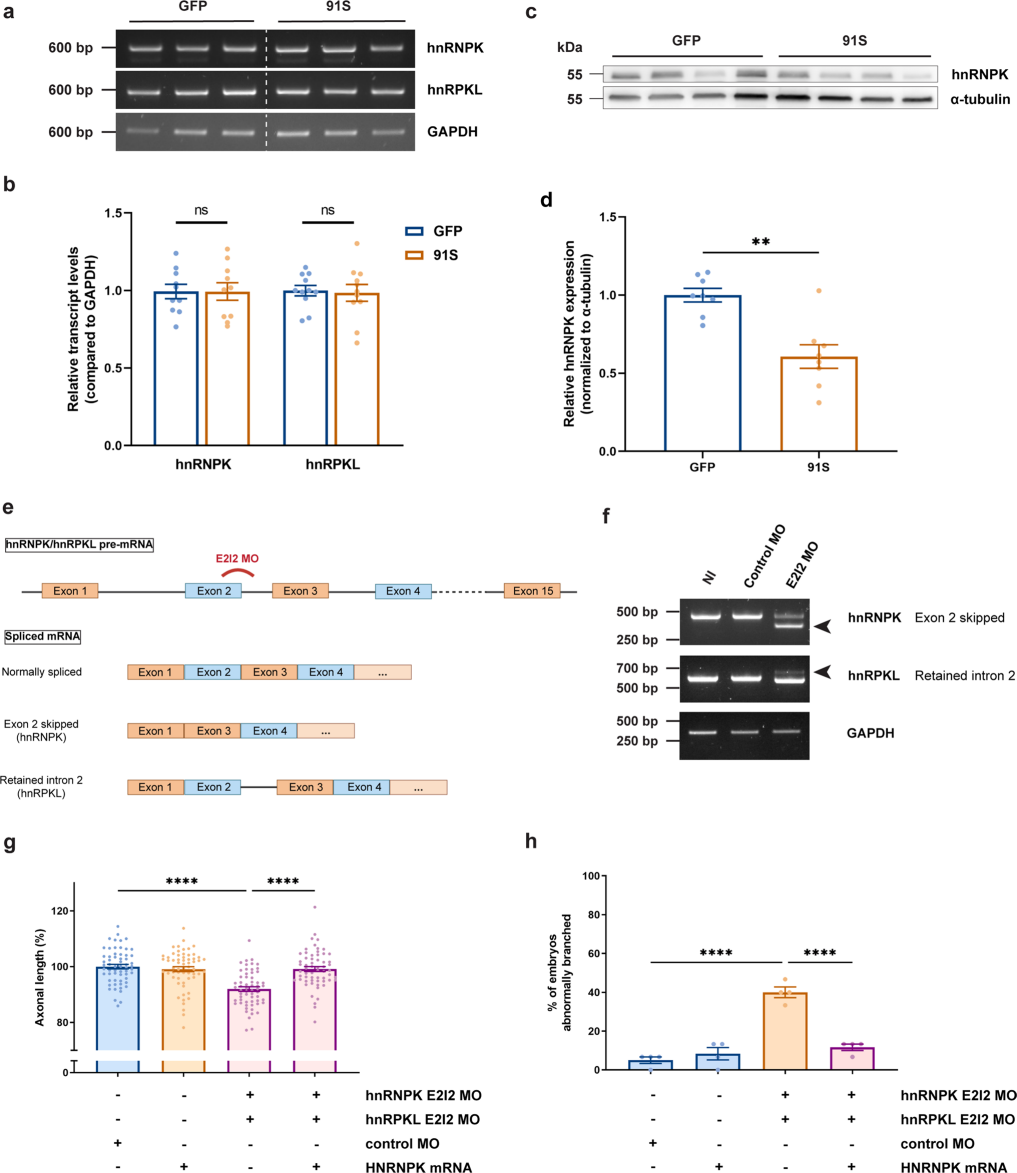
*C9orf72* repeat RNA induces hnRNPK deficiency, which affects motor neuron health in zebrafish embryos. **a** RT-PCR analysis of *HNRNPK* zebrafish orthologues *hnRNPK* and *hnRPKL* in 30 hpf GFP and 91S mRNA-injected zebrafish embryos. **b** Relative quantification of *hnRNPK* and *hnRPKL* transcript levels in 30 hpf GFP and 91S mRNA-injected zebrafish embryos (*N* = 10 experiments). Data represent mean ± SEM. Statistical significance was evaluated with ratio paired *t* test. **c** Western blot detecting endogenous hnRNPK protein levels in 6 hpf GFP and 91S mRNA-injected zebrafish embryos. A-tubulin was used as a loading control. **d** Relative quantification of hnRNPK protein levels in 6 hpf GFP and 91S mRNA-injected zebrafish embryos, normalized to α-tubulin levels (*N* = 8 experiments). Data represent mean ± SEM. Statistical significance was evaluated with ratio paired *t* test; **P < 0.01. **e** Schematic presentation of splice-blocking morpholino targeting the exon 2-intron 2 (E2I2) junction in zebrafish *hnRNPK* and *hnRPKL* pre-mRNA. Outcome of the differentially spliced transcripts is shown. **f** Validation of MO-mediated knockdown by RT-PCR analysis. HnRNPK and hnRPKL splice variants in 30 hpf non-injected (NI), standard morpholino (control MO) and hnRNPK or hnRPKL targeting morpholino (E2I2 MO)-injected embryos are shown. Arrowheads indicate the alternative splice product induced by E2I2 splice-blocking MO. Exon 2 of hnRNPK is skipped and results in a shorter transcript. In hnRPKL, retention of intron 2 results in a higher weight band. GAPDH was used as reference gene. **g**, **h** Effect of morpholino-mediated knockdown of hnRNPK and hnRPKL on axonal length (**g**) and branching (**h**) (*N* = 4 experiments). E2I2 splice-blocking morpholinos were injected at 0.15 mM for hnRNPK and 0.25 mM for hnRPKL. Standard morpholino 0.25 mM. **g**, **h** Data represent mean ± SEM. Statistical significance was evaluated with Kruskal–Wallis test and Dunn’s multiple comparison test (**g**) or one-way ANOVA and Tukey’s multiple comparison test (**h**); ****P < 0.0001

*hnRNPK* and *hnRNPK-like* (or *hnRPKL*) are the zebrafish orthologues of human *HNRNPK*, with 71 and 84% sequence homology, respectively. To determine whether hnRNPK dysfunction contributes to the phenotype in our C9 RNA toxicity zebrafish model, we first assessed the transcript levels of *hnRNPK* and *hnRPKL* by RT-PCR, as well as hnRNPK protein levels by Western blot. We observed that *hnRNPK and hnRPKL* transcript levels remained unaltered in zebrafish (Fig. 2a, b), which was confirmed in iPSC-derived motor neurons and *post-mortem* frontal cortex (Supplementary Fig. 2, online resource) using qPCR and public RNA-sequencing datasets [44, 57, 61]. On the other hand, hnRNPK protein levels were reduced by 40% in zebrafish embryos (Fig. 2c, d), indicating that post-transcriptional regulation of hnRNPK is altered or that its degradation is increased.

Next, we assessed the effect of reduced hnRNPK protein levels on motor neuron development and maintenance. To achieve this, we simultaneously knocked down both *HNRNPK* orthologues in zebrafish embryos using antisense morpholino oligonucleotides (AMOs, referred to as morpholinos) [6, 72]. Splice-blocking morpholinos were used to target the active site of the encoded protein and were designed against the exon 2-intron 2 junction of *hnRNPK* and *hnRPKL* pre-mRNA. Since exon 2 is not a multiple of three, skipping leads to a frameshift in the subsequent amino acid sequence of hnRNPK, while retention of intron 2 (target *hnRPKL*) presumably results in nonsense-mediated decay. After a dose–response experiment (Supplementary Fig. 3a, b, online resource), the effectiveness of these morpholinos was confirmed by RT-PCR (Fig. 2e, f), where a shift in the transcript size confirms efficiency.

Similar to what was observed in the C9 RNA toxicity model, the axonal length of motor neurons was significantly decreased in embryos knocked down for *hnRNPK* and *hnRPKL* compared to embryos injected with standard control morpholino (Fig. 2g). Moreover, 40% of knocked down embryos exhibited abnormally branched motor axons, compared to only 5–10% in control embryos (Fig. 2h). This phenotype was rescued by co-injection of human *HNRNPK* mRNA (Fig. 2g, h), indicating that the effect is HNRNPK-specific and not due to off-target effects. Moreover, this rescue experiment supports the functional conservation of the HNRNPK protein between zebrafish and humans. These data were confirmed by translation-blocking morpholino-mediated knockdown of hnRNPK, specifically targeting the AUG translational start site of both *hnRNPK* and *hnRPKL* (Supplementary Fig. 3c–f, online resource). From these findings, we conclude that hnRNPK is necessary to ensure motor neuron development, that hnRNPK function is disturbed by C9 repeat expansion RNA and that C9 RNA toxicity can be rescued by HNRNPK overexpression.

### C9 RNA toxicity is due to HNRNPK loss-of-function in zebrafish embryos

**Fig. 3:**
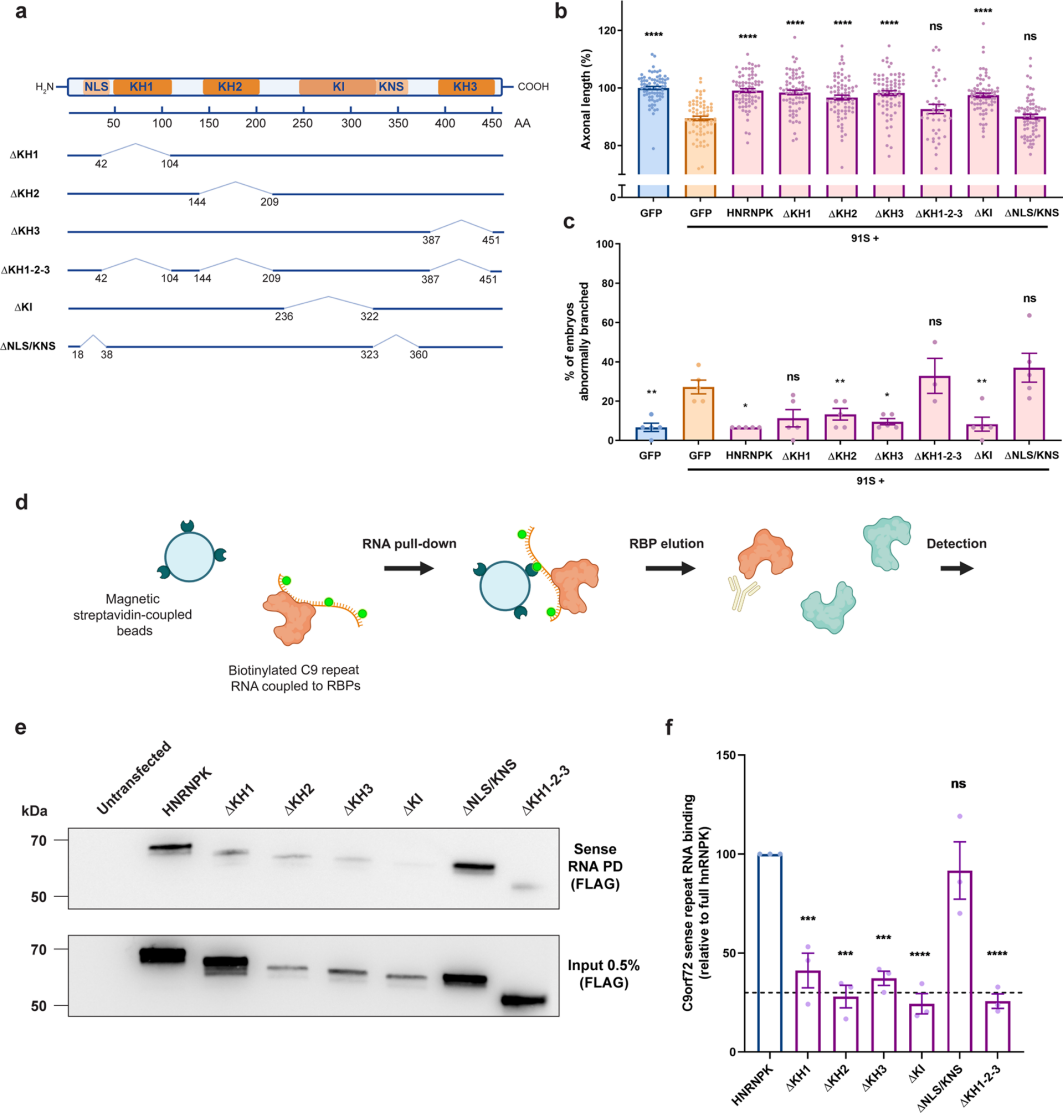
Loss of hnRNPK nuclear localization and RNA binding contributes to the axonopathy in the *C9orf72* RNA toxicity zebrafish model. **a** Schematic model of the HNRNPK protein and its functional domains: RNA-binding domains (KH1, KH2, KH3), protein interaction domain (KI), nuclear localization signal (NLS), nuclear shuttling domain (KNS). Six deletion mutants are illustrated with numbers indicating the position of deleted amino acids (AA) from N- to C-terminus: ∆KH1, ∆KH2, ∆KH3, ∆KH1-2–3, ∆KI, ∆NLS/KNS. Each construct contains a C-terminal FLAG-tag. **b**, **c** Effect of HNRNPK deletion mutant mRNA injections at equimolar dose (0.568 µM) on the 91S (0.844 µM) repeat RNA-induced axonopathy (axonal length (**b**), abnormal branching (**c**)) compared to full-length HNRNPK mRNA injection (0.568 µM) (*N* = 5 experiments). Data represent mean ± SEM. Statistical significance was evaluated with one-way ANOVA and Tukey’s multiple comparison test; *P < 0.05, **P < 0.01, ****P < 0.0001. **d** Schematic workflow of *C9orf72* repeat RNA pull-down assay. In vitro biotinylated *C9orf72* repeat RNA is coupled to repeat RNA-binding proteins upon incubation with HEK cell lysate that is transfected with HNRNPK constructs. Repeat RNA–protein complexes are pulled down using magnetic streptavidin-coupled beads. Detection of bound proteins is done using Western blot. **e** Western blot detecting binding affinity of FLAG-tagged HNRNPK deletion mutant proteins to 91S repeat RNA using FLAG antibody (upper panel). Immunoprecipitation of HEK cells transfected with HNRNPK deletion mutants using FLAG antibody presented as 0.5% input (lower panel). **f** Relative quantification of binding affinity of HNRNPK deletion mutant proteins to 91S repeat RNA compared to the binding affinity of full-length HNRNPK. The dashed line represents the highest level of non-specific binding (*N* = 3 experiments; each data point represents the average of 2 technical replicates). Data represent mean ± SEM. Statistical significance was evaluated with one-way ANOVA and Tukey’s multiple comparison test; ***P < 0.001, ****P < 0.0001

Next, we investigated which functional domains of HNRNPK are essential for its disease-modifying effects. The HNRNPK protein, abundantly expressed and typically localized in the nucleus, contains six functional domains, including three RNA-binding domains (KH1, KH2, KH3), a protein interaction domain (KI) and two nuclear shuttling domains (NLS, KNS) (Fig. 3a) [7]. To investigate the contribution of each domain to the modulation of C9 repeat RNA toxicity, we designed six deletion constructs, each lacking one or multiple functional sites (Fig. 3a). We confirmed the expression of each deletion construct (Supplementary Fig. 4, online resource), and then co-injected 91S repeat RNA with either control *GFP* mRNA, full-length *HNRNPK* mRNA or mRNA encoding one of the six HNRNPK deletion constructs. By comparing the rescue effect of each construct to that of the full size HNRNPK, we were able to determine which domain is essential to rescue the axonal phenotype associated with C9 RNA toxicity in our zebrafish model. Our data showed that removing the protein interaction domain (∆KI) did not alter the modifying effect on the axonal phenotype. On the contrary, the rescue effect of HNRNPK was abolished by deletion of either the nuclear shuttling domains (∆NLS/KNS) or of all RNA-binding domains at once (∆KH1-2-3) (Fig. 3b, c). Interestingly, deletion of individual RNA-binding domains (∆KH1, ∆KH2 or ∆KH3) did not affect the rescue effect, suggesting functional redundancy of these domains.

To ensure that mutant HNRNPK proteins encoded by the deletion constructs are located in the proper subcellular compartments, we assessed their localization in HEK cells by staining against the FLAG-tag expressed on the C-terminal end of each mutant protein (Supplementary Fig. 5, online resource). As expected, HNRNPK lacking nuclear shuttling domains (∆NLS/KNS) exhibited a mainly cytoplasmic localization and was depleted from the nucleus (Supplementary Fig. 5 g, h, online resource). Interestingly, HNRNPK proteins encoded by constructs lacking all RNA-binding domains were also detected in the cytoplasm, in addition to their abundant nuclear presence (Supplementary Fig. 5e, h, online resource). As both of these constructs did not rescue the toxicity of C9 repeat RNA, our data suggest that HNRNPK nuclear localization, as well as RNA binding function, are required for the modifying effect of HNRNPK on C9 repeat RNA toxicity in zebrafish.

HNRNPK was selected as candidate modifying protein because of its known interaction with C9 repeat RNA [13, 25]. To investigate which regions of HNRNPK are responsible for binding to the C9 repeat RNA and to determine the importance of repeat RNA binding in the modifying effect of HNRNPK on RNA toxicity, we performed a repeat RNA pull-down assay using magnetic streptavidin-coupled beads (Fig. 3d). Sense (91S) and antisense (70AS) C9 repeat RNA were biotinylated and used to pull down known RBPs in HEK cell lysates. Endogenous HNRNPK and Purα were pulled down, confirming this interaction [64], while heat shock protein HSP22, used as a negative control, was not (Supplementary Fig. 6a, b, online resource). To test the binding capacity of the deletion constructs of HNRNPK, we performed a similar repeat RNA pull-down experiment on HEK cells transfected with each of the constructs, detecting them via their FLAG-tag. We observed that binding to sense and antisense repeat RNA was significantly decreased for all deletion mutants except for the one lacking the nuclear shuttling domains (∆NLS/KNS) (Fig. 3e, f) (Supplementary Fig. 6c, d, online resource). Since several deletion constructs rescued the motor axonopathy despite decreased C9 repeat RNA binding (i.e., ∆KH1, ∆KH2, ∆KH3 and ∆KI), we conclude that the modifying effect of HNRNPK did not simply rely on interaction with C9 repeat RNA. Indeed, the ∆NLS/KNS deletion construct did not rescue the phenotype, despite the conserved ability to bind C9 repeat RNA. Altogether, these data show that HNRNPK nuclear localization and binding to RNA targets, rather than specific binding to C9 repeat RNA, are crucial for its modifying effect on C9 repeat RNA toxicity.

### Cytoplasmic mislocalization of HNRNPK is a pathological hallmark in C9 ALS patients

**Fig. 4:**
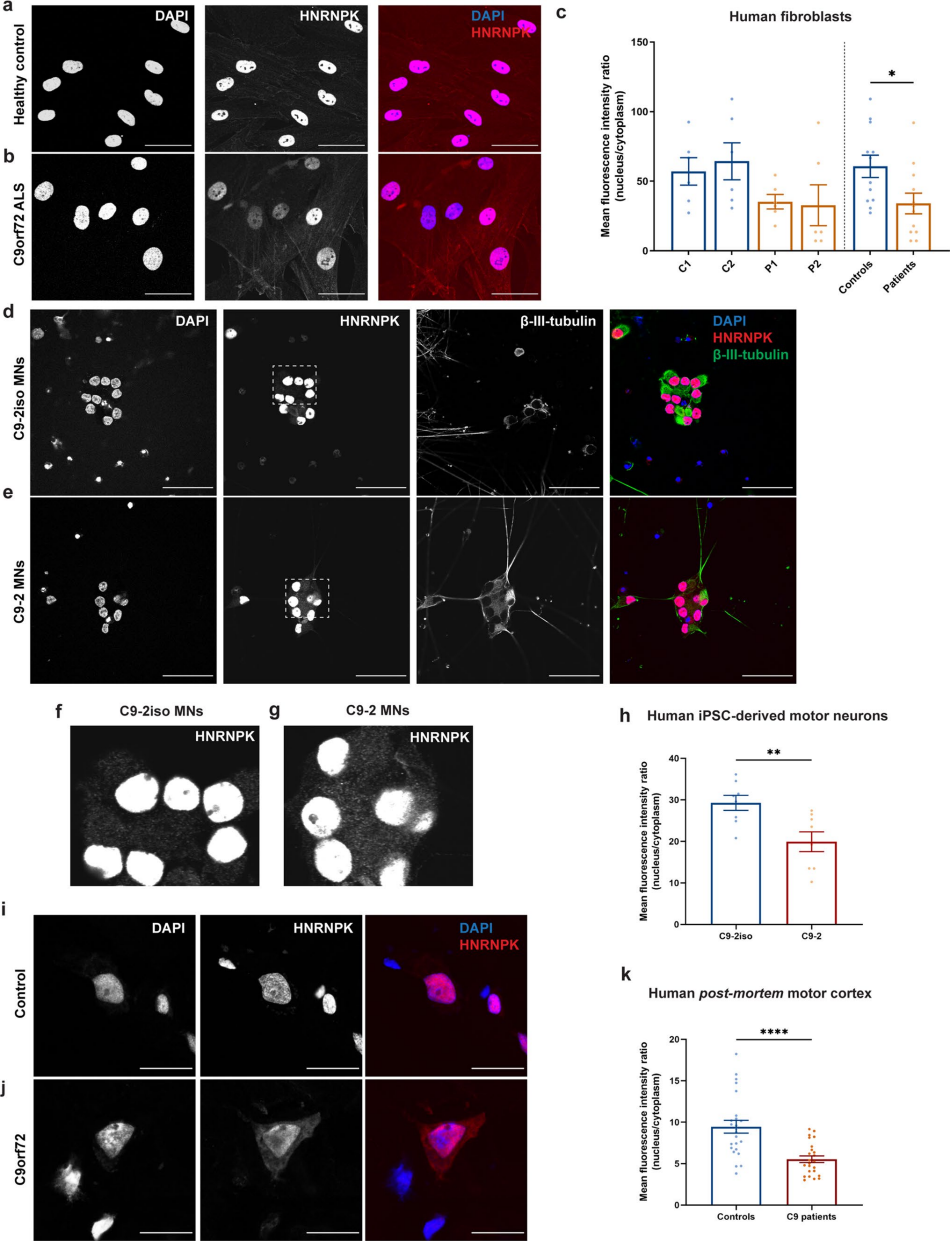
HNRNPK is mislocalized to the cytoplasm in *C9orf72* ALS patient fibroblasts, iPSC-derived motor neurons and ALS patient *post-mortem* motor cortex. **a**, **b** Immunofluorescence of fibroblasts derived from a non-neurodegenerative control and a *C9orf72* ALS patient showing localization of HNRNPK. Scale bar = 50 µm. **c** Quantification of nuclear and cytoplasmic HNRNPK protein levels measured as mean fluorescence intensity ratio (*N* = 3 experiments, 2 technical replicates). Two control fibroblast lines (C1, C2) and two patient lines (P1, P2) were quantified. Pooled control and patient data is indicated on the right side of the dotted line. **d**, **e** Immunofluorescence of mature (38 days) motor neurons derived from iPSCs from a non-neurodegenerative control and a *C9orf72* ALS patient showing localization of HNRNPK. β-III-tubulin was used as a neuronal marker. Scale bar = 50 µm. **f**, **g** Close-up images from (**d**) and (**e**) showing increased cytoplasmic localization of HNRNPK in C9-2 patient line (**g**) compared to its isogenic control (**f**). **h** Quantification of nuclear and cytoplasmic HNRNPK protein levels measured as mean fluorescence intensity ratio (*N* = 4 experiments, 2 technical replicates). One patient line (C9-2) and its corresponding isogenic line (C9-2iso) were quantified. **i**–**k** Immunofluorescence of *post-mortem* motor cortex from a non-neurodegenerative control and a *C9orf72* ALS patient showing localization of HNRNPK. Scale bar = 20 µm. **k** Quantification of nuclear and cytoplasmic HNRNPK protein levels measured as mean fluorescence intensity ratio in 4 non-neurodegenerative controls and 4 C9 ALS/FTD patients (*N* = 3 experiments, 2 technical replicates). **c**, **h**, **k** Data represent mean ± SEM. Statistical significance was evaluated with unpaired *t* test; *P < 0.05, **P < 0.01, ****P < 0.0001. Each data point represents the average N/C ratio per replicate. In total, 10 images were analyzed per experiment and per patient line or sample

As HNRNPK is known to perform its function by binding DNA and RNA targets exclusively in the nucleus, an altered localization in the presence of C9 repeat RNA could induce a loss-of-function of HNRNPK. To determine the exact relevance of loss of HNRNPK function in *C9orf72* ALS, the HNRNPK localization was assessed in three patient-relevant models using immunostainings. First, two *C9orf72* ALS patient fibroblast lines (P1, P2) were compared to two healthy control lines (C1, C2). We observed a > 40% reduction in HNRNPK nucleus/cytoplasm (N/C) ratio, measured as mean fluorescence intensity ratio, due to an increase in cytoplasmic localization of HNRNPK (Fig. 4a-c). Second, induced pluripotent stem cells (iPSCs) derived from a *C9orf72* patient and its corrected isogenic pair [61] were differentiated into spinal motor neurons (sMNs) according to a previously published protocol (Supplementary Fig. 7, online resource) [24]. In this model, we detected an overall N/C reduction of 30% in mature sMNs (Fig. 4d–h). Third, HNRNPK cytoplasmic mislocalization was confirmed in pyramidal neurons in *post-mortem* motor cortex tissue of four C9 ALS/FTD patients compared to four non-neurodegenerative controls (Fig. 4i–k). Overall, three independent patient-derived models provide convincing evidence for HNRNPK cytoplasmic mislocalization in C9 ALS/FTD, which further corroborates the implication of HNRNPK dysfunction in C9 ALS/FTD.

### RRM2 is a downstream transcriptional target of HNRNPK and is dysregulated in *C9orf72* zebrafish

**Fig. 5:**
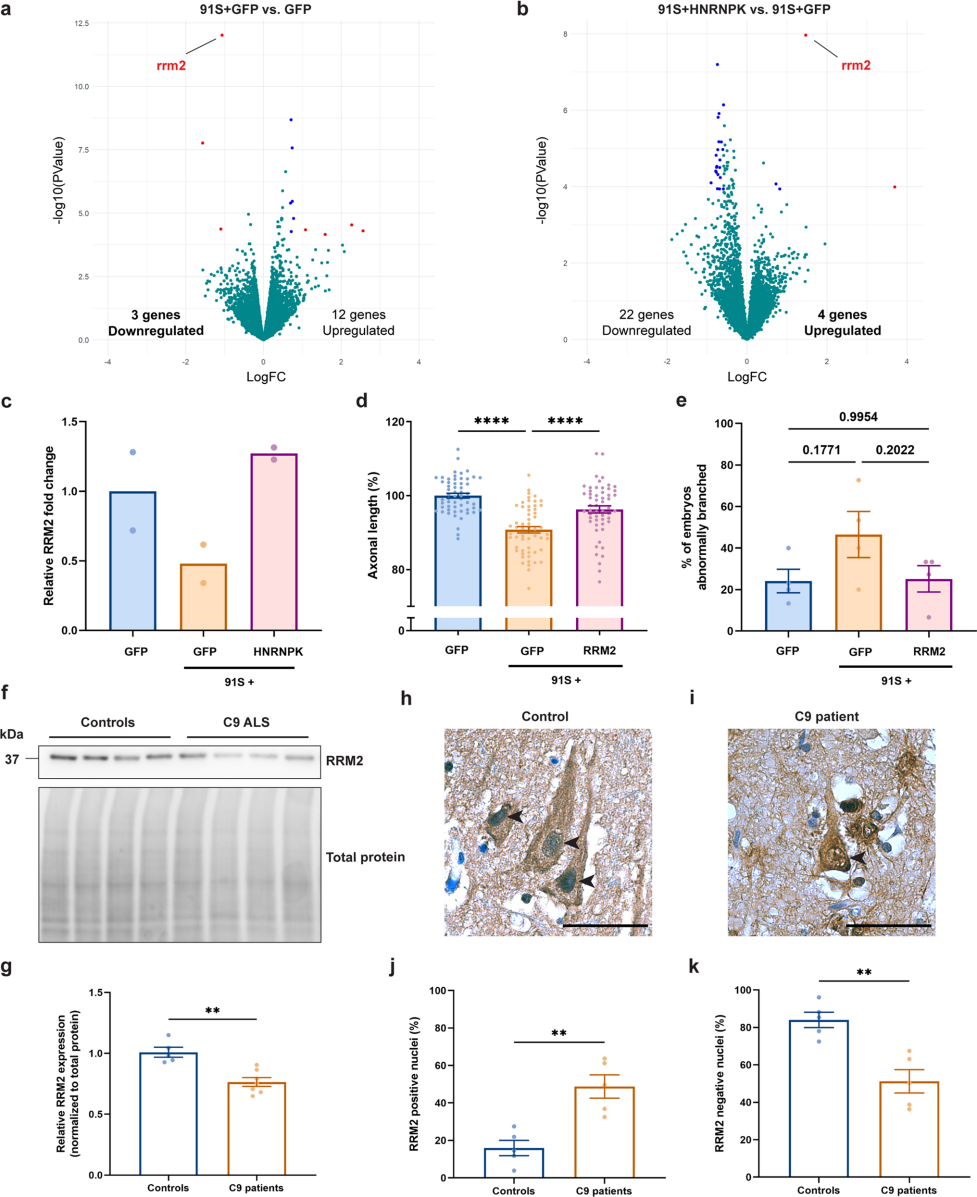
HNRNPK dysfunction induces RRM2 deficiency and nuclear translocation. **a**, **b** Volcano plots showing differentially expressed genes in 30 hpf C9 repeat RNA (91S + GFP) zebrafish embryos compared to GFP control embryos (**a**) and 91S + HNRNPK- compared to 91S + GFP-injected embryos (**b**). Significant (*P* < 0.0001) up- or down-regulated genes with a logFC > 1 or < − 1 and a logFC > 0.5 or < − 0.5 are indicated in red and blue respectively. Turquoise dots represent all non-significant differentially expressed genes. RRM2 transcripts are downregulated in C9 repeat RNA zebrafish embryos (**a**) and are upregulated upon overexpression of HNRNPK (**b**). **c** Bar graph showing the relative RRM2 fold change (*N* = 2 biological replicates). **d**, **e** Effect of *RRM2* mRNA injection (0.314 µM) on the 91S repeat RNA-induced axonopathy on axonal length (**d**) and abnormal branching (**e**) (*N* = 4 experiments). Data represent mean ± SEM. Statistical significance was evaluated with one-way ANOVA and Tukey’s multiple comparison test;****P < 0.0001. *P* values are indicated for comparison of abnormal branching. **f** Western blot detecting RRM2 protein levels in *post-mortem* motor cortex of non-neurodegenerative controls and *C9 ALS/FTD*. Total protein was used to normalize data. **g** Relative quantification of RRM2 protein levels in 5 non-neurodegenerative controls and 7 C9 patients. Data represent mean ± SEM. Statistical significance was evaluated with unpaired *t* test; **P < 0.01. (**h**, **i**) Immunohistochemical detection of RRM2 in motor cortex of a representative non-neurodegenerative control (**h**) and a *C9orf72* ALS (**i**) case. Arrowheads indicate nuclei of neuronal cells stained negative (**h**) or positive (**i**) for RRM2. Scale bar = 50 µm. **j**, **k** Percentage of cells containing nuclei that stain positive (**j**) and negative (**k**) for RRM2 in motor cortex of 5 non-neurodegenerative controls and 5 C9 patients. Data represent mean ± SEM. Statistical significance was evaluated with unpaired *t* test; **P < 0.01

To further investigate the HNRNPK dysfunction in the context of C9 repeat RNA toxicity, we assessed dysregulated gene expression to identify downstream HNRNPK targets. To achieve this, we performed transcriptome-wide analysis using bulk RNA-sequencing of 30 hpf zebrafish embryos injected with control *GFP* mRNA and 91S repeat RNA co-injected with *GFP* mRNA (91S + GFP). Statistical analysis showed that three genes were significantly downregulated, while 12 genes were upregulated (Fig. 5a). The strongest hit as shown in the volcano plot was *rrm2,* the zebrafish orthologue of r*ibonucleotide reductase regulatory subunit M2 (RRM2*), of which a 0.5-fold reduced expression was detected in the C9 repeat RNA toxicity zebrafish model compared to *GFP* mRNA control-injected embryos (Fig. 5a, c). By comparing HNRNPK overexpression (91S + HNRNPK) with the C9 RNA toxicity model (91S + GFP), we discovered four significantly upregulated genes, with *rrm2* again being the strongest hit (Fig. 5b, c), suggesting that this upregulation could be involved in the rescue effect of HNRNPK. This hypothesis is strengthened by the identification of *RRM2* as a transcriptional target of HNRNPK in a large-scale RBP CLIPseq study released at the ENCODE Data Coordination Center (https://www.encodeproject.org) and obtained from the Gene Expression Omnibus (GSE91621) [74].

To investigate the importance of RRM2 in the modifying mechanism on C9 RNA toxicity, we tested whether overexpression of RRM2 was sufficient to rescue the axonopathy caused by 91S repeat RNA in our zebrafish model. Therefore, we injected mRNA encoding human RRM2 together with C9 repeat RNA in 1- to 2-cell stage zebrafish oocytes after confirmation of its expression (Supplementary Fig. 1f, online resource) and determination of the highest non-toxic dose (Supplementary Fig. 1 g, h, online resource). Assessment of SV2-stained motor axons showed a significant increase in axonal length and a trend of a decrease in aberrant branching when compared to embryos only injected with 91S repeat RNA (Fig. 5d, e), demonstrating that RRM2 overexpression could indeed rescue the phenotype. Altogether, our results indicate that RRM2 is a downstream target of HNRNPK, which is dysregulated in *C9orf72* ALS and is involved in the C9 repeat RNA toxicity mechanism.

### *C9orf72* ALS patients display RRM2 depletion and nuclear translocation

To determine whether RRM2 expression and functionality is altered and, if so, which aspect is affected in C9 ALS/FTD, we first performed a Western blot using *post-mortem* motor cortex tissue and compared RRM2 protein levels in C9 ALS/FTD patients with those in non-neurodegenerative controls. Consistent with an expected decrease in RNA transcripts, as observed in our zebrafish model, we noted a 30% reduction in RRM2 levels in *C9orf72* patients (Fig. 5f, g). RRM2 is responsible for catalyzing the formation of deoxyribonucleotides in DNA synthesis. In particular, higher expression and nuclear translocation (i.e., activation) of RRM2 is associated with DNA replication and repair processes [14]. Therefore, we performed immunohistochemistry on non-neurodegenerative control and *C9orf72* patient motor cortex tissue in order to investigate the activation status of RRM2. Neuronal nuclei were visually inspected and subsequently scored positive or negative for RRM2. We observed significantly more staining in nuclei of pyramidal motor neurons, located in layer V of the motor cortex, of *C9orf72* patients compared to non-neurodegenerative controls (Fig. 5h–k). Nuclear translocation of RRM2 was also observed in spinal cord of C9 ALS patients, but not in occipital cortex (Supplementary Fig. 8, online resource), suggesting that primarily the motor neuron-containing tissues display RRM2 alterations. Altogether, these data show changes in expression and localization of RRM2, which strongly indicates a disturbed function of RRM2 in C9 ALS/FTD.

### HNRNPK downregulation or dysfunction, associated with reduced RRM2 levels, results in increased DNA damage

Since the cells primarily affected in ALS/FTD are post-mitotic neurons, disturbed DNA replication is unlikely to be the driving force of RRM2 dysregulation. Both HNRNPK and RRM2 are also involved in the DNA damage response although, their interaction at this level is not characterized [14, 50, 77]. To explore the link between these proteins in the context of DNA damage, we investigated the cellular response to stress when HNRNPK is downregulated and when DNA damage is induced [75].

**Fig. 6:**
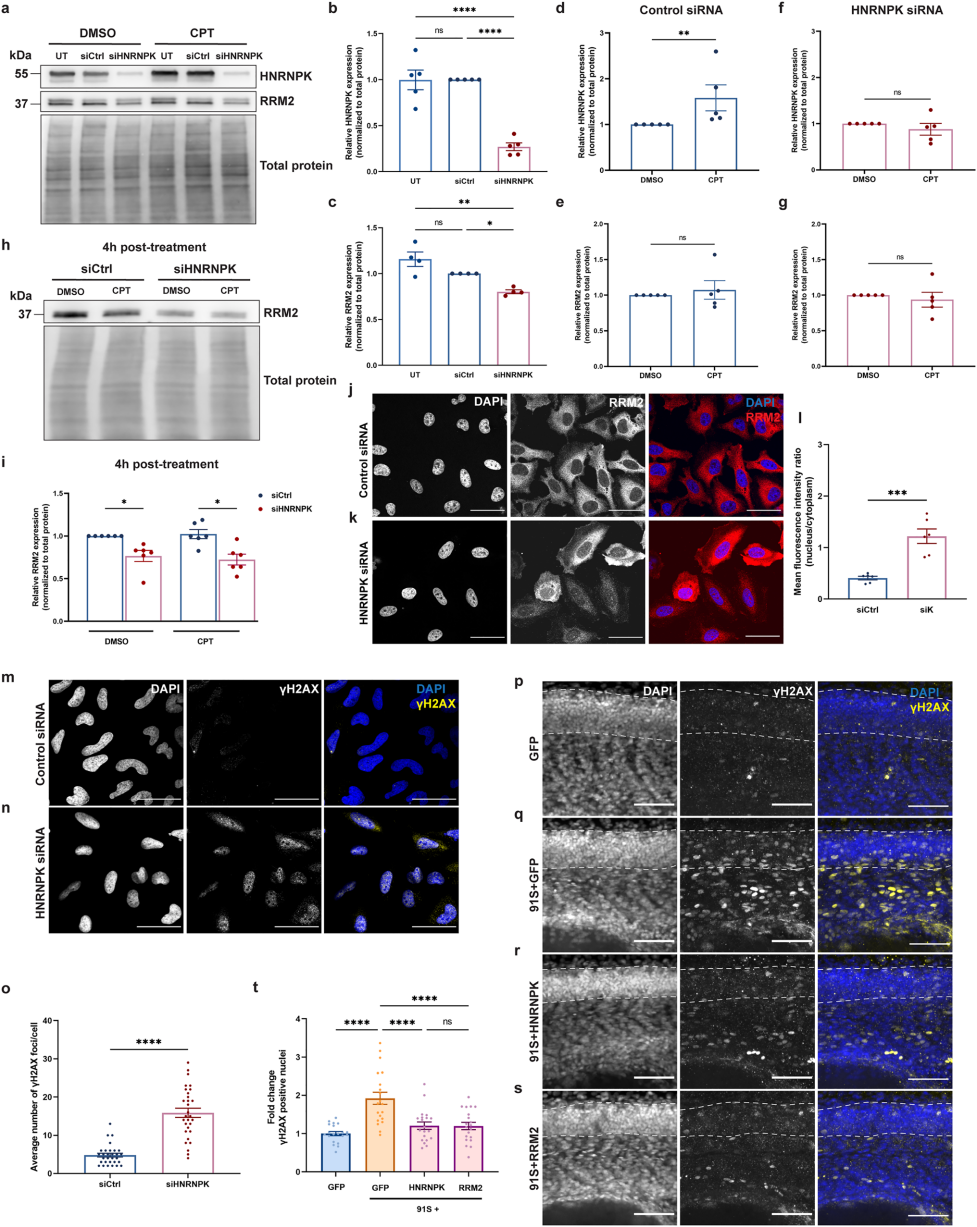
HNRNPK and RRM2 are implicated in the DNA damage response in C9 RNA toxicity. **a** Western blot detecting HNRNPK and RRM2 levels. The upper panel shows the confirmation of reduced HNRNPK protein levels in HeLa cells transfected with HNRNPK siRNA and treated with DMSO or 10 µM CPT. In the middle panel, RRM2 protein levels in untransfected, control siRNA (siCtrl)- and HNRNPK siRNA (siHNRNPK)-transfected cells are presented. Total protein staining was used as loading control (lower panel). **b**, **c** Quantification of HNRNPK (**b**) and RRM2 (**c**) protein levels in untransfected CPT-treated cells, or in cells transfected with siCtrl or siHNRNPK (*N* = 4–5 experiments). **d**–**g** Quantification of HNRNPK (**d, f**) and RRM2 levels (**e**, **g**) in siCtrl- (**d**, **e**) or siHNRNPK-transfected cells (**f**, **g**) treated with DMSO or CPT (*N* = 5 experiments). **h** Western blot detecting reduced RRM2 levels in siHNRNPK-transfected cells compared to cells transfected with siCtrl and collected 4 h post-treatment (upper panel). No difference is observed between DMSO- and CPT-treated cells. Total protein staining was used as loading control (lower panel). **i** Quantification of RRM2 protein levels (*N* = 6 experiments). **j**, **k** Immunostaining of RRM2 in siCtrl- (**j**) and siHNRNPK-transfected cells (**k**). **l** Quantification of nuclear and cytoplasmic RRM2 protein levels measured as mean fluorescence intensity ratio (*N* = 3 experiments, 2 technical replicates). Each data point represents the average N/C ratio per replicate. In total, 10 images were analyzed per experiment and per condition. Scale bar = 50 µm. **m**, **n** Immunostaining of γH2AX foci in siCtrl- (**m**) and siHNRNPK-transfected (**n**) HeLa cells. Scale bar = 50 µm. **o** Quantification of the average number of γH2AX foci per cell (*N* = 3 experiments). **p**–**s** Immunostaining of γH2AX foci in GFP (**p**), 91S + GFP (**q**), 91S + HNRNPK (**r**) and 91S + RRM2 (**s**) RNA-injected 30 hpf zebrafish embryos. Dotted lines indicate the borders of the spinal cord. Scale bar = 50 µm. **t** Quantification of the fold change of γH2AX positive nuclei in the spinal cord of zebrafish embryos (*N* = 3 experiments). **b**–**g**, **i**, **l**, **o**, **t** Data represent mean ± SEM. Statistical significance was evaluated with one-way ANOVA and Tukey’s multiple comparison test (**b**, **c**, **t**), unpaired *t* test (**d**–**g**, **l**) or Kruskal–Wallis test and Dunn’s multiple comparison test (**i**, **o**); *P < 0.05, **P < 0.01, ***P < 0.001, ****P < 0.0001

We first performed an siRNA-mediated knockdown of HNRNPK in HeLa cells. Upon transfection of siRNA against HNRNPK, there was a 80% decrease in protein levels when compared to the control siRNA condition (Fig. 6a, b). This was accompanied by a 20% decrease in RRM2 levels (Fig. 6a, c), confirming the link between RRM2 and HNRNPK. A second approach was to assess the cell response to DNA damage induced by the topoisomerase I inhibitor camptothecin (CPT). Interestingly, when treated with CPT, HNRNPK protein levels were significantly increased in control siRNA-transfected cells as compared to a vehicle treatment (Fig. 6a, d). When HNRNPK was reduced by siRNA, CPT had no clear additional effect on HNRNPK levels (Fig. 6a, f). Remarkably, CPT treatment did not alter RRM2 protein levels despite changes in the HNRNPK levels (Fig. 6a, b), either immediately (Fig. 6a, e, g) or 4 h post-treatment (Fig. 6h, i). This indicates that upregulation of RRM2 expression, as would be expected based on our bulk RNA-sequencing the zebrafish HNRNPK rescue model (Fig. 5b), is not involved in the early phase of the DNA damage response. As we did not find changes in RRM2 protein levels, we postulated that CPT treatment could instead lead to its activation. We subsequently assessed its localization in HeLa cells and in line with previous studies [54, 79], we confirmed that DNA damage induced the nuclear translocation of RRM2, which was detected 4 h post CPT-treatment (Supplementary Fig. 9, online resource). Interestingly, we found that a reduction in HNRNPK via siRNA was sufficient to induce translocation of RRM2 to the nucleus (Fig. 6j–l). This shows that depletion of HNRNPK, like CPT treatment, triggers the DNA damage response resulting in the nuclear migration of RRM2.

In order to quantify DNA damage, we stained HeLa cells with an antibody against γH2AX, a phosphorylated histone variant representing an early response to DNA damage, and counted the number of γH2AX foci per cell, each representing one DNA double-strand break [39]. We observed that knockdown of HNRNPK was sufficient to increase γH2AX foci (Fig. 6m–o), and that treatment with CPT resulted in a similar number of γH2AX foci per cell (Supplementary Fig. 10a–d, g, online resource). Treated cells that were also depleted of HNRNPK showed a more pronounced increase in punctae (Supplementary Fig. 10e–g, online resource). Overall, our results indicate a mechanistic link between HNRNPK, RRM2 and DNA damage, suggesting that in our model, DNA damage cannot be efficiently repaired due to reduced RRM2 expression downstream of disrupted HNRNPK.

### C9 RNA toxicity induces DNA damage, which is rescued with HNRNPK or RRM2

Finally, to demonstrate the link between C9 RNA toxicity and DNA damage, we assessed DNA damage in C9 repeat RNA-injected zebrafish embryos. In particular, we counted the number of neuronal nuclei that were positive for γH2AX in the spinal cord of 30 hpf zebrafish embryos and observed a twofold increase in zebrafish injected with C9 repeat RNA compared to *GFP* control embryos (Fig. 6p, q, t). Strikingly, the addition of *HNRNPK* or *RRM2* mRNA prevented the increase in γH2AX foci (Fig. 6r, s, t). Overall, our data indicate that C9 RNA toxicity induces DNA damage and that the functionality of HNRNPK, primarily through the transcriptional regulation of its downstream target RRM2, is essential for maintaining an operational DNA damage response.

## Discussion

In this study, we explored the relevance of C9 RNA toxicity as a disease mechanism in C9 ALS/FTD and investigated modifiers using a target RBP known to bind C9 repeat RNA [12, 25]. We discovered that HNRNPK, a member of the heterogeneous nuclear ribonucleoproteins (HNRNPs), is able to specifically modify C9 repeat RNA toxicity in a zebrafish model. Interestingly, the beneficial effect on the phenotype, namely altered spinal motor neuron axonal length and branching, was similar to what was previously observed for another modifier, the transcription activator Purα [64]. In addition, we found that the nuclear localization and RNA-binding domains were essential for the modifying effect of HNRNPK. These findings, together with the observed cytoplasmic mislocalization of HNRNPK in patient models suggested HNRNPK loss-of-function as a pathological event in *C9orf72* ALS pathophysiology. We then identified ribonucleotide reductase regulatory subunit M2 (RRM2) as a downstream transcriptional target of HNRNPK, and found that its function is altered by repeat RNA-induced dysfunction of HNRNPK. We discovered that RRM2 expression is reduced in *C9orf72* ALS, and found that RRM2 is mechanistically linked with HNRNPK in the DNA damage response pathway.

The *C9orf72* embryonic zebrafish model represents an acute toxic phenotype, rather than progressive neurodegeneration, as in *C9orf72* ALS patients [64]. In this regard, the motor axonal phenotype observed in 30 hpf embryos was used as a screening phenotype, in which we could identify modifiers of pure RNA toxicity, without the contribution of DPRs. Even though we cannot claim that this model is representative of the overall pathogenesis of *C9orf72* ALS, it encompasses a consistent and reproducible motor axonopathy induced by *C9orf72* repeat RNA. The purpose of including patient-relevant models (i.e., *C9orf72* patient fibroblasts and iPSC-derived motor neurons) and *post-mortem* material in this study is to enable translation of the embryonic zebrafish screening to *C9orf72* ALS in the clinic, and to assess the relevance of HNRNPK as a modifier of RNA toxicity in disease. However, investigation of DPR-induced displacement of HNRNPK, which has already been associated with other RBPs [30], will be required to reveal whether or not RNA and DPR toxicity are mutually exclusive in *C9orf72* ALS.

Processing of mRNAs, including alternative splicing and transcriptional as well as translational regulation, is the overall purpose of HNRNPs [22]. HNRNPs have already been linked with ALS in the context of disease-associated missplicing, and for their role in the downstream stress response and DNA damage [3]. More specifically, some HNRNPs were shown to be involved not only in general ALS mechanisms, such as the HNRNP family member and aggregation-prone TAR DNA-binding protein 43 (TDP-43) [58], but also to be relevant in familial ALS, including HNRNPA3 in *C9orf72* ALS [47].

HNRNPK is one of the most abundantly expressed HNRNP members and is widely distributed across tissues affected in ALS, such as the cerebral cortex, spinal cord and muscle [67, 76]. The RNA metabolism of several genes is regulated in the nuclear compartment by HNRNPK [37, 76]. Recently, RNA-sequencing of myocytes derived from sporadic ALS patient cells has shown a disease-associated downregulation of the *HNRNPK* gene [38]. Moreover, biochemical analysis and immunohistochemical stainings revealed lowered HNRNPK expression in patient spinal cord tissue and fibroblasts from sporadic ALS and familial TDP-43 ALS cases [48, 49], whereas our data and publically available datasets indicated unaltered transcript levels in *C9orf72* ALS patient iPSC-derived motor neurons and frontal cortex [44, 57, 61]. Similarly, in our zebrafish model for C9 RNA toxicity, we observed decreased HNRNPK levels, however, without changes at the transcript level. Translational impairments, enhanced protein degradation or repeat RNA sequestration-related detection issues might explain the overall inconsistency between transcript and protein levels. C9 repeat RNA leads to sequestration of a wide range of RBPs, conceivably including some RBPs that also regulate the translation of HNRNPK, which can impair the overall process to a greater extent. To illustrate this, the RNA-binding protein YBX1 is a regulator of HNRNPK [27, 63] and is part of the *C9orf72* repeat RNA interactome [12, 25], which indicates the sequestration of YBX1 in RNA foci. Hence, a lack of translation, rather than increased degradation, is more conceivable to be at the origin of reduced HNRNPK expression. Furthermore, it is not yet known whether altered HNRNPK expression, or solely the loss of its function due to sequestration or mislocalization, is involved in the pathogenesis of C9 ALS/FTD. However, both of these situations lead to less available HNRNPK, which is detrimental for the DNA damage response.

To investigate a change in function, we used HNRNPK deletion constructs and discovered that nuclear localization and RNA binding of HNRNPK, independent from its interaction with C9 repeat RNA, are necessary for the observed modification of the phenotype associated with C9 RNA toxicity in our zebrafish model. To evaluate the overall relevance of HNRNPK localization in *C9orf72* ALS, we used two independent patient models (i.e., *C9orf72* ALS patient fibroblasts, iPSC-derived motor neurons) and *post-mortem* tissue to demonstrate that HNRNPK localization is mislocalized to the cytoplasm in *C9orf72* ALS, as was recently shown for FTD [4]. Each model or disease sample has its own characteristics and represents a different stage in the disease, most likely accompanied with a specific phase of HNRNPK mislocalization, which can explain the differences in altered localization. Various factors could have instigated this mislocalization. First, an aberrant increase in cytoplasmic intron-retaining transcripts was reported in ALS [68], and as they are predicted to bind to RBPs they could also contribute to the observed mislocalization of HNRNPK. Second, osmotic stress is known to be sufficient for the disease-associated cytoplasmic localization of ALS-linked RBPs, including TDP-43, FUS, HNRNPA1 as well as of HNRNPK [26]. Third, the phosphorylation status of HNRNPK determines its subcellular localization to a certain extent and has been linked with TDP-43 aggregation [48]. Aside from the abovementioned mechanisms linking ALS with RBP mislocalization, we hypothesized that a *C9orf72*-specific mechanism could be at play, as HNRNPK mislocalization in *C9orf72* ALS could be instigated by the direct interaction with cytoplasmic RNA foci and/or soluble repeat RNA. This is in line with findings in the C9 RNA toxicity zebrafish model displaying solely cytoplasmic, and not nuclear, RNA foci [64]. While only few reports detected cytoplasmic C9 repeat RNA foci [12, 13, 42, 58], the presence of soluble cytoplasmic repeat RNA in *C9orf72* ALS patients could also be sufficient to bind and sequester HNRNPK, preventing its translocation to the nucleus where it performs its function. Crucially, evidence of pathogenicity for RNA foci remains limited, suggesting that the soluble repeat RNA might be more toxic than the RNA foci themselves, as was originally thought. Nevertheless, the exact contribution of these factors to the interplay of HNRNPK with ALS-related actors and its mislocalization remains elusive and warrants further investigation.

Recently, HNRNPK mislocalization was reported to induce splicing dysregulation of downstream genes in the frontal cortex of FTD patients [4]. We, therefore, searched for impaired HNRNPK effectors in C9 ALS/FTD using datasets and in vivo, cellular, as well as patient-derived models, and identified RRM2 as a novel downstream target of HNRNPK. RRM2 is responsible for catalyzing synthesis of deoxyribonucleotides for the purpose of DNA synthesis for cell proliferation [14], and with relevance to neurodegenerative diseases, RRM2 is required for DNA damage repair through delivering deoxyribonucleotides to the sites of DNA damage [14, 79]. Likewise, HNRNPK has been shown to be involved in DNA damage and has been broadly studied in the context of cancer research [21, 76, 77]. As transcription of oncogenes p53 and c-Myc are known to be regulated by HNRNPK [34, 50, 51, 56], this further substantiates the involvement of DNA damage-related actors, and a role for HNRNPK in general *C9orf72* ALS. Moreover, this increases the likelihood that HNRNPK specifically regulates *RRM2* transcription, in addition to its RNA binding or modification as indicated by the published CLIP-seq datasets. To confirm the mechanistic link between HNRNPK and DNA damage in our model, we found that in absence of HNRNPK, HeLa cells showed increased DNA damage. We then demonstrated that upregulation of HNRNPK expression was triggered as a response to induced DNA damage by treating the cells with CPT. These findings imply that the downstream mechanism of HNRNPK dysfunction occurs within the same cell type. However, on top of the impaired DNA damage response observed in one single cell type, we cannot exclude non-cell autonomous effects in the context of a multi-cellular model or in *C9orf72* ALS patients. We found that RRM2 protein levels were reduced and that its cell distribution was altered towards an increasing nuclear localization, both in C9 ALS/FTD patient samples and upon HNRNPK depletion in HeLa cells. The use of RRM2 agonists, antagonists [62] or drugs indirectly leading to a change in its expression [35, 36] could provide novel insights in the relevance of impaired DNA damage repair in C9 and more general ALS.

Over the past years, arginine-rich DPRs produced in C9 ALS/FTD have been associated with many cellular deficits leading to neurodegeneration, including a failed response to DNA damage [2]. Increased HNRNPA3 levels have been associated with reduced RNA foci and toxic arginine-rich DPRs (i.e., polyPR and polyGR), which have been shown to increase the frequency of DNA double strand breaks [47, 53]. Moreover, loss of nuclear HNRNPA3 was demonstrated to increase DPR levels (i.e., polyGA), leading to accumulation of DNA damage [53]. Notably, p53 reduction was shown to rescue axonal degeneration induced by C9 DPR toxicity [41]. However, because no clear distinction was made between RNA and DPR toxicity in the abovementioned models, it is not possible to conclude on their individual contribution to overall DNA damage in C9 ALS/FTD. Here, we found that the availability of functional HNRNPK, and accordingly functional RRM2, is sufficient to prevent neurodegeneration specifically caused by C9 RNA toxicity, without the involvement of DPR toxicity. Our results strongly indicate that failure in mounting an appropriate ‘DNA damage response’ (DDR) as a response to repeat RNA toxicity is particular to ALS/FTD harboring *C9orf72* repeat expansions.

**Fig. 7:**
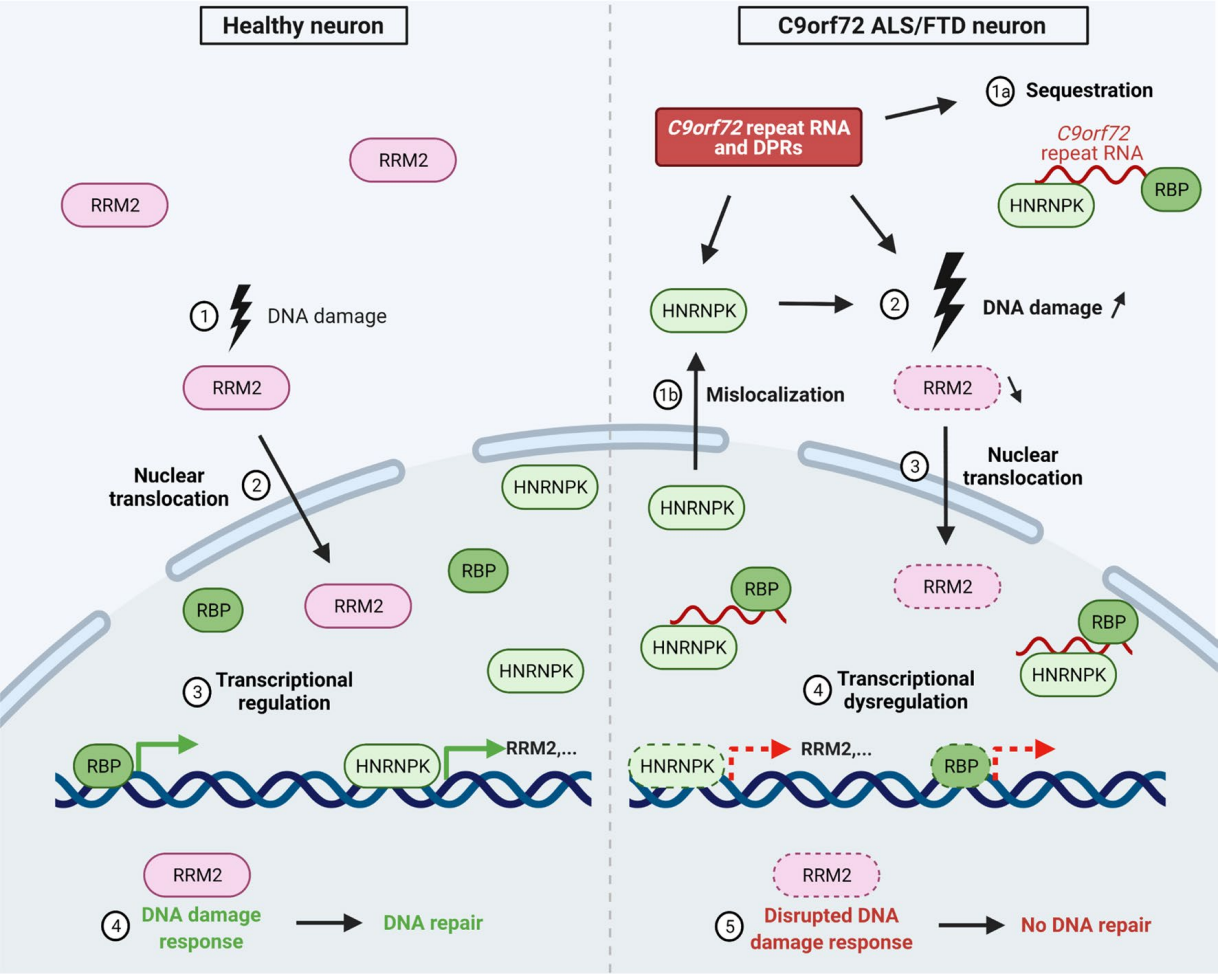
Schematic model linking mechanistic insights of HNRNPK and RRM2 in *C9orf72* ALS. In the left panel, event of naturally occurring DNA damage (1) in a healthy neuron with consequential RRM2 activation and nuclear translocation (2). HNRNPK is a transcriptional regulator of RRM2 (3), essential for DNA repair in the DNA damage response (4). In the right panel, a neuron affected in C9 ALS/FTD is characterized by DPRs and RNA foci, consisting of *C9orf72* repeat RNA and sequestered RNA-binding proteins, including HNRNPK (1a). Next to sequestration, HNRNPK is mislocalized to the cytoplasm (1b), resulting in a loss-of-function of HNRNPK, and directly or indirectly increasing DNA damage, which activates RRM2 (3). Loss-of-function achieves transcriptional dysregulation of downstream effectors of HNRNPK, including RRM2 (4). Activated, though depleted RRM2 results in a disrupted DNA damage response, impeding DNA repair (5). Scheme created with BioRender.com

Overall, our working model (Fig. 7) illustrates how changes in HNRNPK expression or activity are involved in C9 ALS/FTD. Firstly, HNRNPK is mislocalized prior to, next to, or as a consequence of sequestration by C9 repeat RNA into RNA foci, which could by itself increase DNA damage. Second, unavailable or disrupted HNRNPK leads to downstream transcriptional dysregulation of, amongst others, RRM2. Initiation of DNA damage repair mechanisms triggers RRM2’s activation and nuclear translocation, but reduced expression prevents successful DNA repair. DNA damage repair gets increasingly important during the aging process. Therefore, small changes in the maintenance mechanisms can lead to progressive motor neuron degeneration, having an impact on disease progression in C9 ALS/FTD.

In conclusion, using in vivo and in vitro models we identified HNRNPK as a novel modifying protein of RNA toxicity in *C9orf72* ALS. We hypothesize that HNRNPK loss-of-function, either by lack of proper expression or mislocalization, contributes to pathogenicity in *C9orf72* ALS. Furthermore, we demonstrated that a disrupted DNA damage response is attributable to dysfunctional HNRNPK, and that the subsequent RRM2 deficiency leads to impairments in DNA repair in the C9 RNA toxicity model. Altogether, our results highlight RNA toxicity as an undervalued mechanism that at least partially, and perhaps concomitant with DPR toxicity, contributes to neurodegeneration in C9 ALS/FTD. As related studies further illustrated that the altered DNA damage response is a recurring mechanism at play in other ALS/FTD types, such as TDP-43 ALS [28, 29], our work points to HNRNPK and, in particular, its target RRM2 as novel focuses in developing new therapeutic strategies for ALS.

## Supplementary Information

Below is the link to the electronic supplementary material.

Supplementary file1 (PDF 1893 KB)

Supplementary file2 (XLSX 11 KB)

Supplementary file3 (XLSX 18 KB)

Supplementary file4 (XLSX 25 KB)
