# Supplementary material for: HNRNPK alleviates RNA toxicity by counteracting DNA damage in C9orf72 ALS
Source: Acta Neuropathol. 2022 Jul 27;144(3):465–88. doi: 10.1007/s00401-022-02471-y (PMC9381635; doi:10.1007/s00401-022-02471-y)
Supplement: Supplementary file 1 — Supplementary file1 (PDF 1893 KB) [file 401_2022_2471_MOESM1_ESM.pdf]

Electronic supplementary material

HNRNPK alleviates RNA toxicity by counteracting DNA damage in *C9orf72* ALS

Elke Braems[1,2], Valérie Bercier[1,2*], Evelien Van Schoor[1,2,3], Kara Heeren[1,2], Jimmy Beckers[1,2], Laura Fumagalli[1,2], Lieselot Dedeene[1,2,3,4], Matthieu Moisse[1,2], Ilse Geudens[1,2], Nicole Hersmus[1,2], Arpan R. Mehta[5,6], Bhuvaneish T. Selvaraj[5,6], Siddharthan Chandran[5,6], Ritchie Ho[7], Dietmar R. Thal[3,8], Philip Van Damme[1,2,9], Bart Swinnen[1,2], Ludo Van Den Bosch[1,2*]

[1] KU Leuven - University of Leuven, Department of Neurosciences, Experimental Neurology and Leuven Brain Institute (LBI), Leuven, Belgium

[2] VIB, Center for Brain & Disease Research, Laboratory of Neurobiology, Leuven, Belgium

[3] KU Leuven - University of Leuven, Department of Imaging and Pathology, Laboratory of Neuropathology and Leuven Brain Institute (LBI), Leuven, Belgium

[4] KU Leuven - University of Leuven, Department of Neurosciences, Laboratory for Molecular Neurobiomarker Research, Leuven Brain Institute (LBI), Leuven, Belgium.

[5] University of Edinburgh, UK Dementia Research Institute, Edinburgh, UK

[6] University of Edinburgh, Centre for Clinical Brain Sciences, Edinburgh, UK

[7] Cedars-Sinai Medical Center, Board of Governors Regenerative Medicine Institute, Los Angeles, USA

[8] University Hospitals Leuven, Department of Pathology, Leuven, Belgium

[9] University Hospitals Leuven, Department of Neurology, Leuven, Belgium

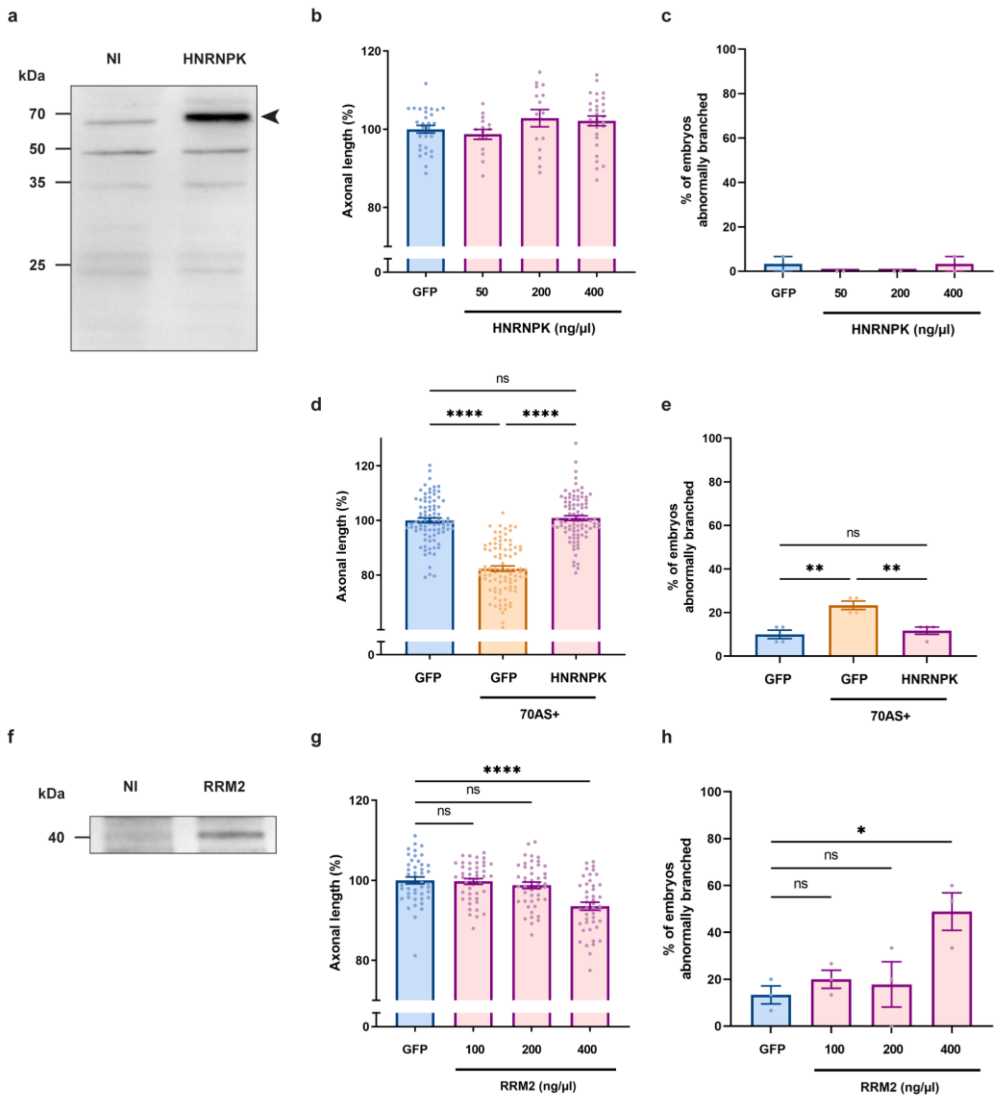

Supplementary Fig.1 Confirmation of HNRNPK expression and dose-dependent toxicity in zebrafish embryos. **(a)** Western blot using FLAG antibody confirming expression of HNRNPK in 6 hpf zebrafish embryos upon overexpression of HNRNPK mRNA. Arrowhead indicates the band specific for HNRNPK. **(b,c)** Toxicity screen of HNRNPK (50 ng/µl (0.071 µM), 100 ng/µl (0.142 µM), 200 ng/µl (0.284 µM), 400 ng/µl (0.568 µM)) compared to GFP control condition (0.568 µM) on axonal length **(b)** and branching **(c)** in 30 hpf zebrafish embryos (N = 2 experiments; n = 15-30 embryos analyzed per condition). **(d,e)** Alleviating effect of HNRNPK mRNA injection (0.568 µM) on the 70AS repeat RNA-induced axonopathy, showing increased axonal length **(d)** and reduced abnormal branching **(e)** (N = 4 experiments). **(f)** Western blot using FLAG antibody detecting expression of FLAG-tagged RRM2 in 6 hpf zebrafish embryos injected with RRM2 mRNA. **(g,h)** Toxicity screen of RRM2 (100 ng/µl (0.157 µM), 200 ng/µl (0.314 µM), 400 ng/µl (0.629 µM)) compared to GFP control condition (0.629 µM) on axonal length **(g)** and branching **(h)** in 30 hpf zebrafish embryos (N = 3 experiments; n = 45 embryos analyzed per condition). **(b-e,g,h)** Data represent mean ± SEM. Statistical significance was evaluated with one-way ANOVA and Tukey's multiple comparison test; *P<0.05, **P<0.01, ****P<0.0001.

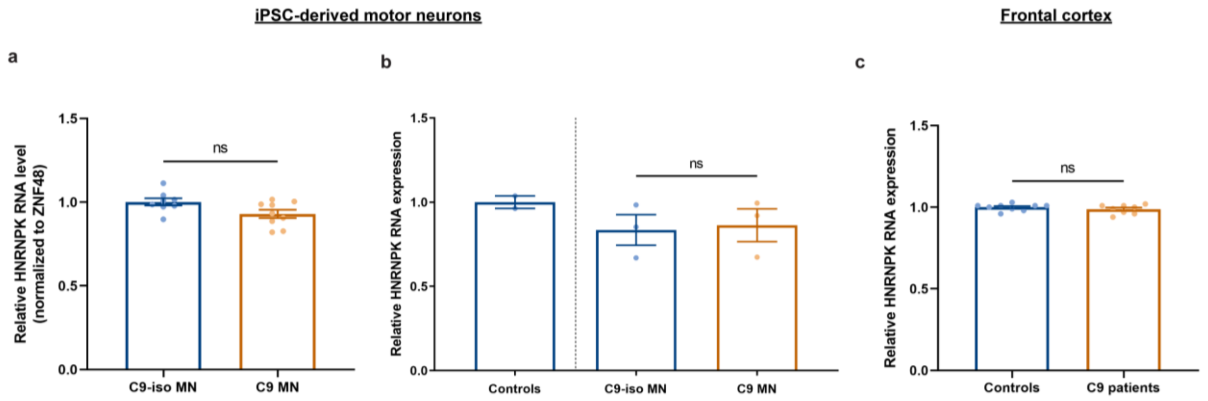

Supplementary Fig.2 *HNRNPK* transcript levels remain unaltered in *C9orf72* iPSC-derived motor neurons and frontal cortex. (a) qPCR detection of *HNRNPK* transcript levels, normalized to *ZNF48*, in iPSC-derived motor neurons of a C9 ALS patient and its isogenic control line (N = 8). **(b)** Relative RNA expression level of *HNRNPK* in iPSC-derived motor neurons from unaffected controls, *C9orf72* patients and their isogenic control lines, based on the published RNA-sequencing datasets from Selvaraj *et al.* [3] and Mehta *et al.* [1]. **(c)** Relative RNA expression level of *HNRNPK* in frontal cortex tissue from 9 unaffected controls and 8 *C9orf72* ALS patients, based on the published RNA-sequencing dataset from Prudencio *et al.* [2]. Data represent mean ± SEM. Statistical significance was evaluated with **(a,c)** unpaired t test and **(b)** ratio paired t test.

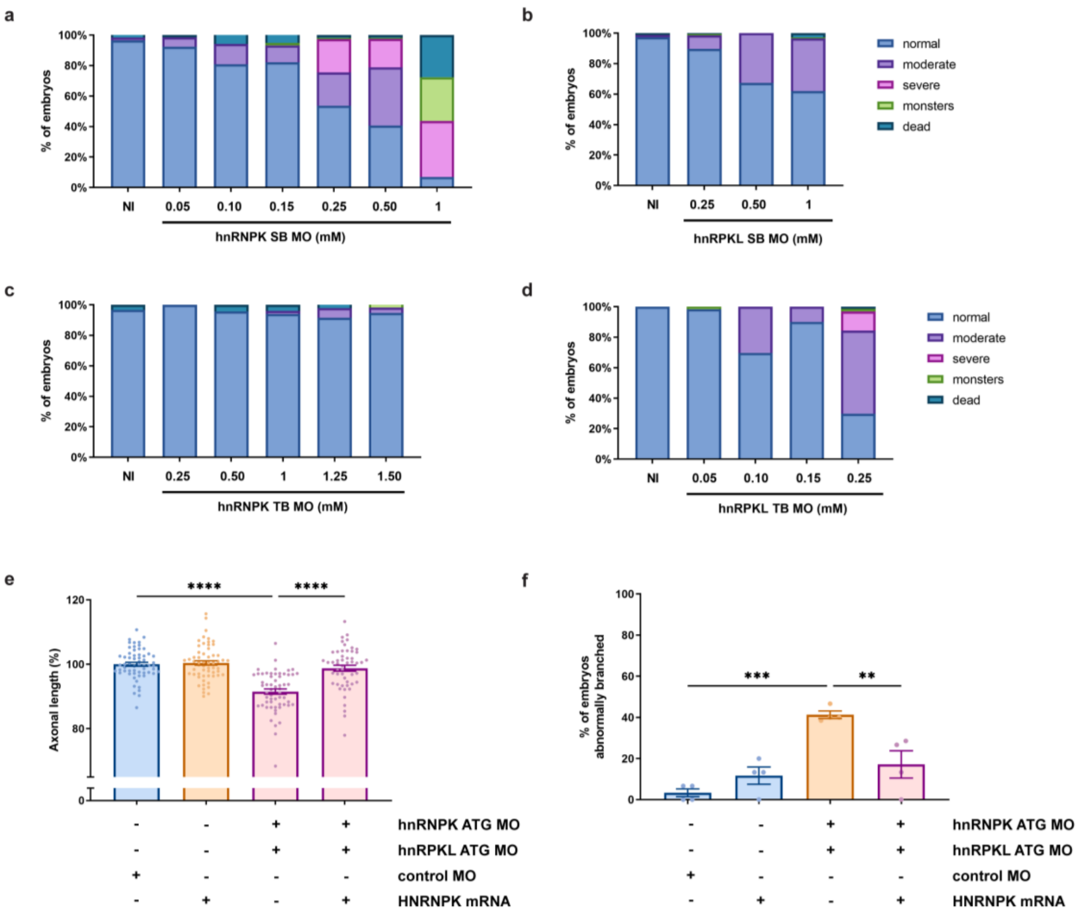

Supplementary Fig.3 Dose responses of hnRNPK/L morpholinos and confirmation of the role of hnRNPK/L in neuronal health using translation-blocking morpholinos. (a-d) Morphological analysis of 30 hpf zebrafish embryos injected with different doses of splice-blocking (SB) **(a,b)** and translation-blocking (TB) **(c,d)** morpholinos targeting hnRNPK **(a,c)** and hnRPKL **(b,d)**. Doses: hnRNPK SB MO, 0.05-0.10-0.15-0.25-0.50-1 mM (n = 87-172 embryos) **(a)**; hnRPKL SB MO, 0.25-0.50-1 mM (n = 92-152 embryos) **(b)**; hnRNPK TB MO, 0.25-0.50-1-1.25-1.50 mM (n = 43-60 embryos) **(c)**; hnRPKL TB MO, 0.05-0.10-0.15-0.25 mM (n = 59-66 embryos) **(d)**. **(e,f)** Effect of morpholino-mediated knockdown of hnRNPK and hnRPKL on axonal length **(e)** and branching **(f)** (N = 4 experiments). Translation-blocking morpholinos were injected at 1.50 mM for hnRNPK and 0.10 mM for hnRPKL. Standard morpholino 1.50 mM. **(e,f)** Data represent mean ± SEM. Kruskal-Wallis test and Dunn's multiple comparison test **(e)** or one-way ANOVA and Tukey's multiple comparison test **(f)**; **P<0.01, ***P<0.001, ****P<0.0001.

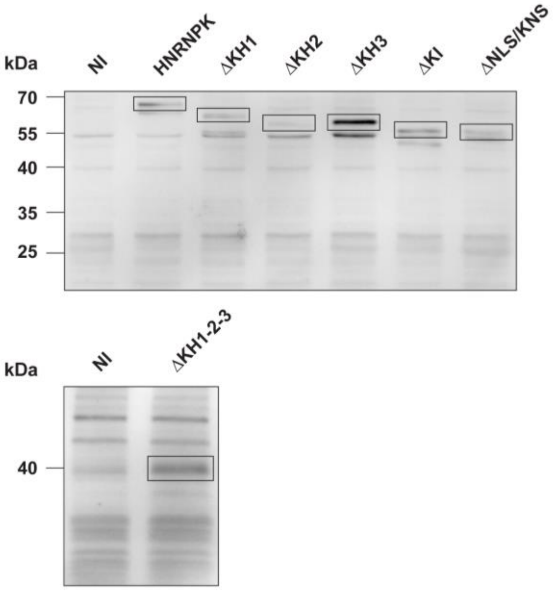

Supplementary Fig.4 Expression of HNRNPK deletion mutants in zebrafish embryos. Western blot using FLAG antibody confirming expression of HNRNPK deletion mutants in 6 hpf zebrafish embryos upon overexpression of HNRNPK deletion construct mRNA. Boxes indicate the band specific for each HNRNPK deletion mutant.

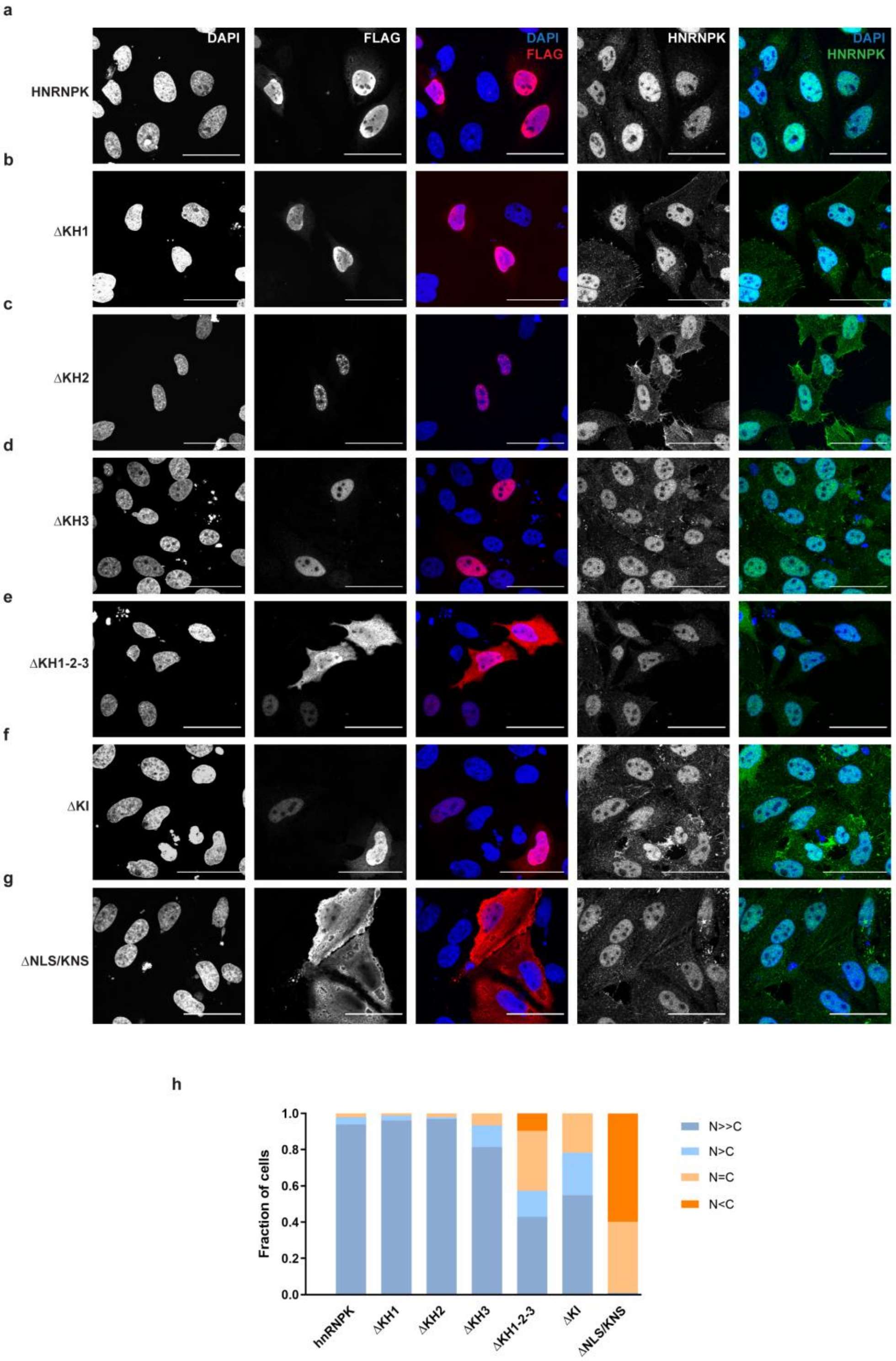

Supplementary Fig.5 Localization of HNRNPK deletion mutants in HEK cells. (a-g) Immunostaining of endogenous HNRNPK and FLAG-tagged HNRNPK deletion mutants (full-length HNRNPK **(a)**, ΔKH1 **(b)**, ΔKH2 **(c)**, ΔKH3 **(d)**, ΔKH1-2-3 **(e)**, ΔKI **(f)**, ΔNLS/KNS **(g)**) overexpressed in HEK cells using FLAG and HNRNPK antibody. Scale bar = 50 µm. **(h)** Stacked column graphs presenting the percentage of cells containing HNRNPK mutant proteins which are mostly nuclear (N>>C), more nuclear than cytoplasmic (N>C), equally nuclear and cytoplasmic (N=C) or more cytoplasmic than nuclear (N<C).

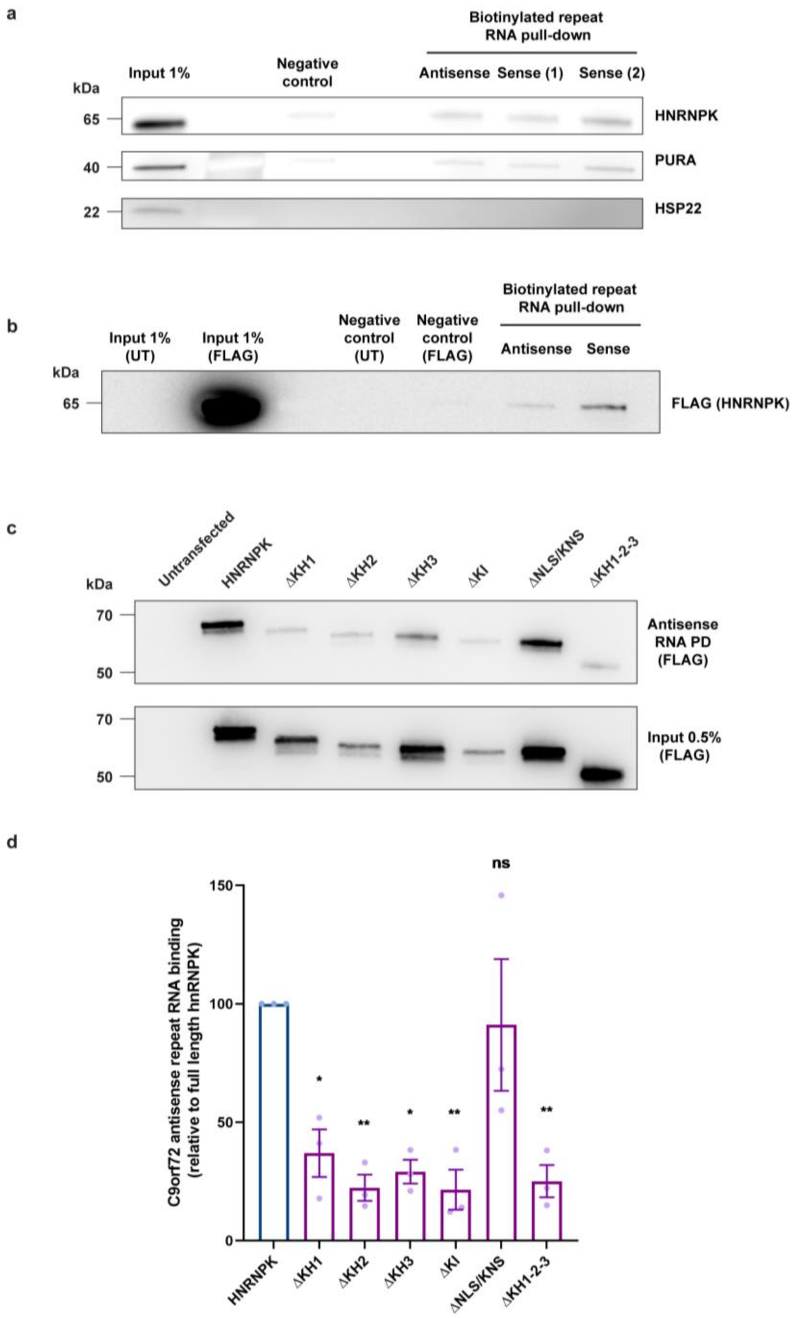

Supplementary Fig.6 Endogenous and transfected FLAG-tagged HNRNPK in HEK cells is pulled down by sense and antisense C9 repeat RNA. (a,b) Western blot detecting binding affinity of endogenous HNRNPK **(a)** or FLAG-tagged HNRNPK **(b)** to antisense (70AS) and sense (91S) C9 repeat RNA using HNRNPK **(a)** or FLAG **(b)** antibody. Immunoprecipitation of HEK cells untransfected (UT) or transfected with FLAG-tagged HNRNPK is presented as 1% input. The negative control lanes represent pulled-down samples without the addition of repeat RNA**.** Detection of PURA **(a – second panel)** and HSP22 **(a – third panel)** was done as a positive and negative binding control respectively. **(c)** Western blot detecting binding affinity of FLAG-tagged HNRNPK deletion mutant proteins to 70AS (antisense) repeat RNA using FLAG antibody (upper panel). The input was loaded at 0.5% of total lysate. **(d)** Relative quantification of binding affinity of HNRNPK deletion mutant proteins to 70AS repeat

RNA compared to the binding affinity of full-length HNRNPK (N = 3 experiments; each data point represents the average of 2 technical replicates). Data represent mean ± SEM. Statistical significance was evaluated with one-way ANOVA and Tukey's multiple comparison test; *P<0.05, **P<0.01.

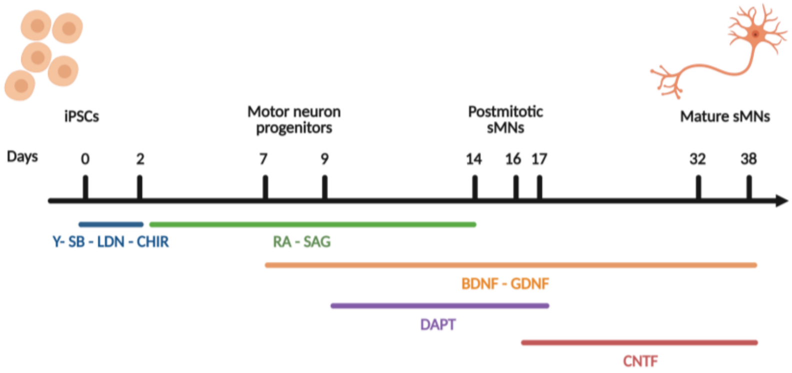

Supplementary Fig.7 Differentiation protocol of C9 iPSC-derived motor neurons. Schematic overview of the motor neuron differentiation protocol. Experiments were performed on mature motor neurons, at day 38 of differentiation.

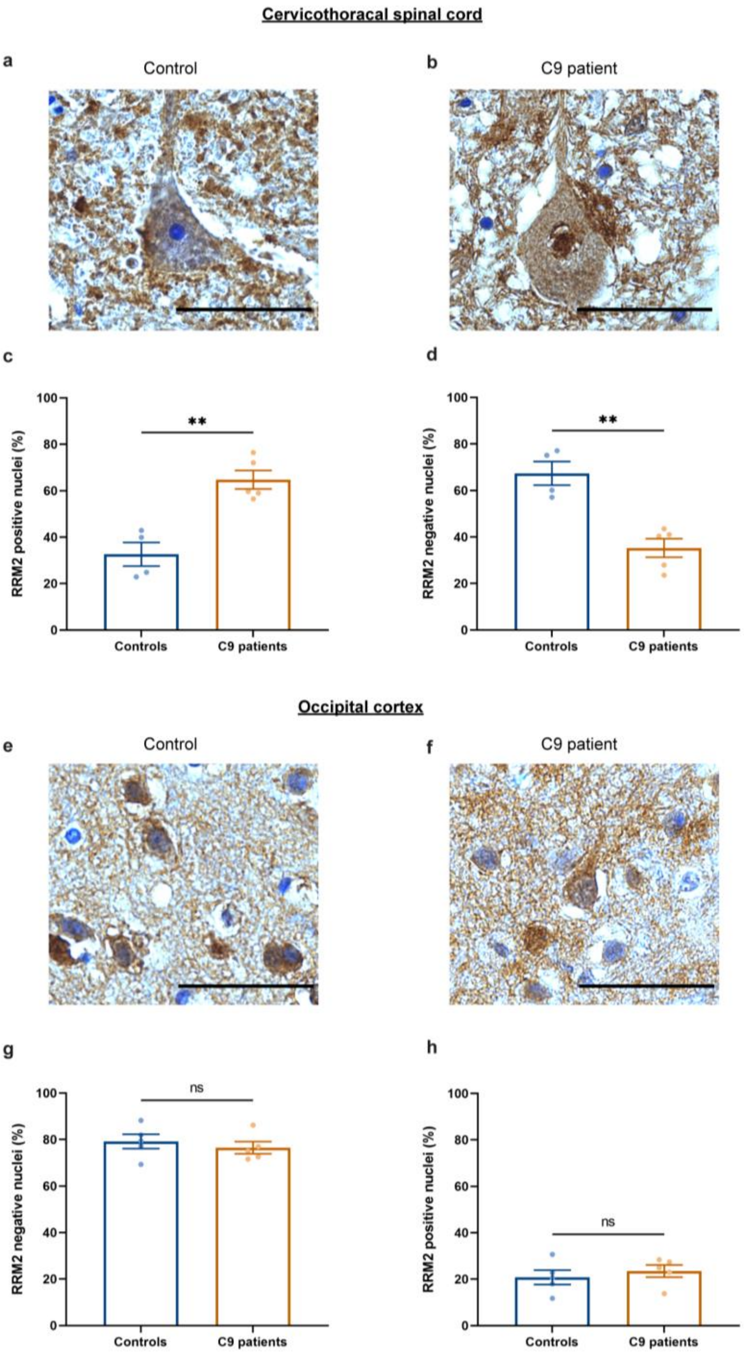

Supplementary Fig.8 RRM2 is translocated to the nucleus in C9 patient spinal cord, but not in occipital cortex. Immunohistochemical detection of RRM2 in cervicothoracal spinal cord **(a-d)** and occipital cortex **(e-h)** of a representative non-neurodegenerative control **(a,e)** and a *C9orf72* ALS **(b,f)** case. Scale bar = 50 µm. Percentage of cells containing nuclei that stain positive **(c,g)** and negative **(d,h)** for RRM2 in spinal cord **(c,d)** and occipital cortex **(g,h)** of 4-5 non-neurodegenerative controls and 5 C9 patients. Data represent mean ± SEM. Statistical significance was evaluated with unpaired t test; **P<0.01.

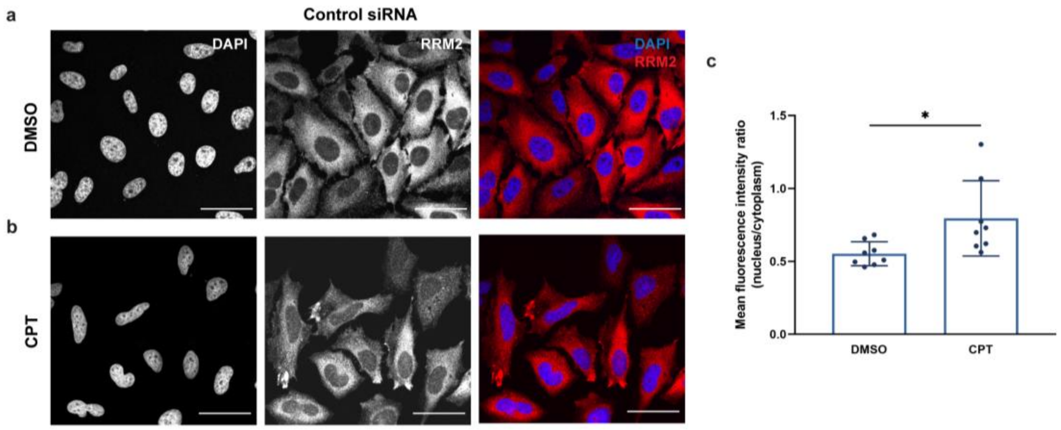

Supplementary Fig.9 DNA damage induces translocation of RRM2 to the nucleus. (a,b) Immunostaining of RRM2 in DMSO-treated **(a)** and 10 µM CPT-treated **(b)** cells. Control siRNA-transfected cells were treated for 30 min and collected 4 h post-treatment. Scale bar = 50 µm. **(c)** Quantification of nuclear and cytoplasmic RRM2 protein levels measured as mean fluorescence intensity ratio (N = 4 experiments, 2 technical replicates). Data represent mean ± SEM. Statistical significance was evaluated with Mann-Whitney U test; *P<0.05. Each data point represents the average N/C ratio per replicate. In total, 10 images were analyzed per experiment and per condition.

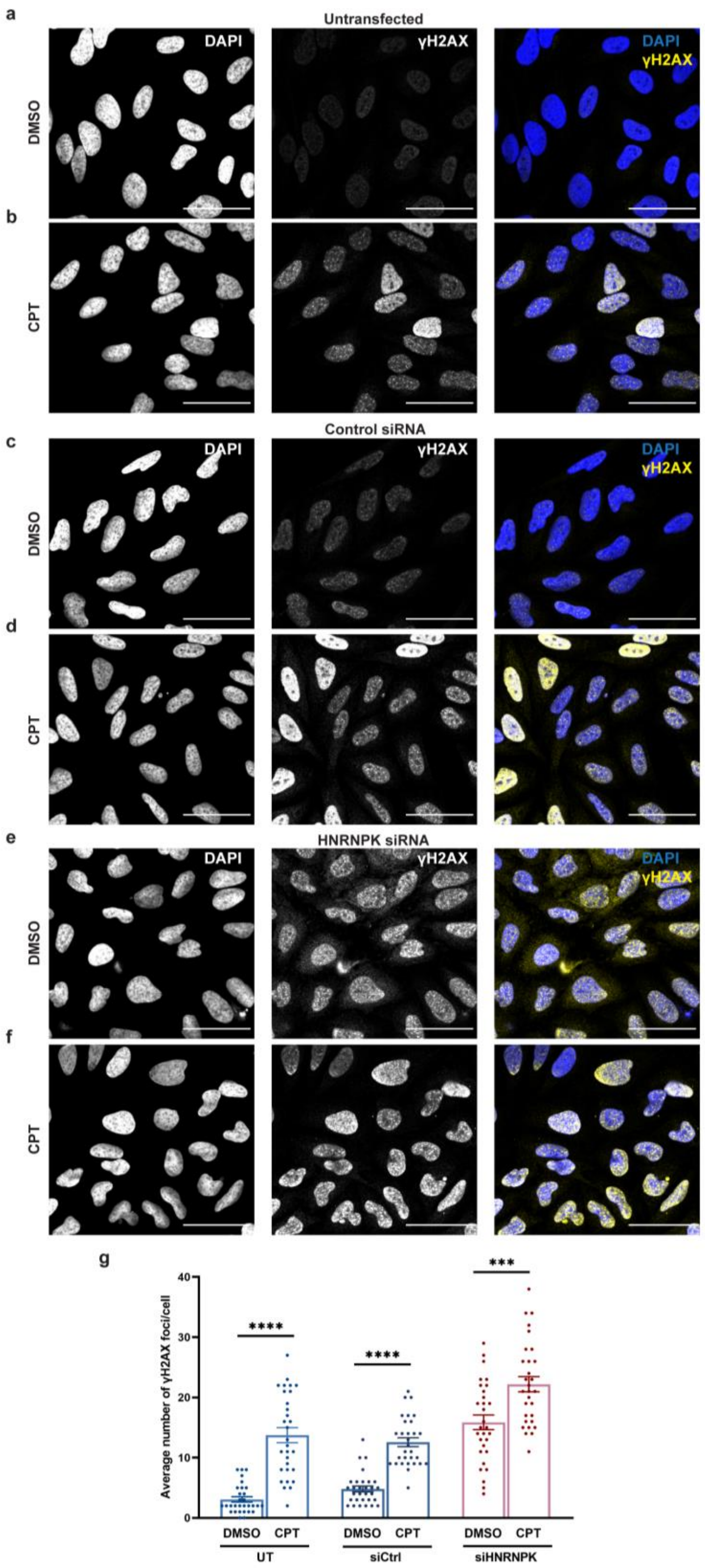

Supplementary Fig.10 DNA damage in camptothecin-treated HeLa cells. Immunostaining of γH2AX foci in DMSO-treated **(a,c,e)** and 10 µM CPT-treated **(b,d,f)** HeLa cells. 30 min treatment was performed on untransfected cells **(a-b)** or on 72 h post-transfected cells with control siRNA **(c-d)** or HNRNPK siRNA **(e-f)**. Scale bar = 50 µm. **(g)** Quantification of the average number of γH2AX foci per cell in untransfected, control siRNA and HNRNPK siRNA transfected cells.(N = 3 experiments) Data represent mean ± SEM. Statistical significance was

evaluated with unpaired t test (for UT and siHNRNPK-transfected cells) or Mann-Whitney U test (for siCtrl-transfected cells); ***P<0.001, ****P<0.0001.

References

1. Mehta AR, Gregory JM, Dando O, Carter RN, Burr K, Nanda J, Story D, McDade K, Smith C, Morton NM, Mahad DJ, Hardingham GE, Chandran S, Selvaraj BT (2021) Mitochondrial bioenergetic deficits in C9orf72 amyotrophic lateral sclerosis motor neurons cause dysfunctional axonal homeostasis. Acta Neuropathol 141:257–279. doi: 10.1007/s00401-020-02252-5

2. Prudencio M, Belzil V V., Batra R, Ross CA, Gendron TF, Pregent LJ, Murray ME, Overstreet KK, Piazza-Johnston AE, Desaro P, Bieniek KF, DeTure M, Lee WC, Biendarra SM, Davis MD, Baker MC, Perkerson RB, Van Blitterswijk M, Stetler CT, Rademakers R, Link CD, Dickson DW, Boylan KB, Li H, Petrucelli L (2015) Distinct brain transcriptome profiles in C9orf72-associated and sporadic ALS. Nat Neurosci 18:1175–1182. doi: 10.1038/nn.4065

3. Selvaraj BT, Livesey MR, Zhao C, Gregory JM, James OT, Cleary EM, Chouhan AK, Gane AB, Perkins EM, Dando O, Lillico SG, Lee YB, Nishimura AL, Poreci U, Thankamony S, Pray M, Vasistha NA, Magnani D, Borooah S, Burr K, Story D, McCampbell A, Shaw CE, Kind PC, Aitman TJ, Whitelaw CBA, Wilmut I, Smith C, Miles GB, Hardingham GE, Wyllie DJA, Chandran S (2018) C9ORF72 repeat expansion causes vulnerability of motor neurons to Ca2+-permeable AMPA receptor-mediated excitotoxicity. Nat Commun 9:347. doi: 10.1038/s41467-017-02729-0
